# Single-bubble EHD behavior into water two-phase flow under electric-field stress and gravitational acceleration using PFM

**DOI:** 10.1038/s41526-021-00134-y

**Published:** 2021-02-18

**Authors:** Maryam Aliakbary Mianmahale, Arjomand Mehrabani-Zeinabad, Masoud Habibi Zare, Mahdi Ghadiri

**Affiliations:** 1 Isfahan University of Technology, Department of Chemical Engineering, 84156-83111 Isfahan, Iran; 2 Institute of Research and Development, Duy Tan University, Da Nang, 550000 Viet Nam; 3 The Faculty of Environment and Chemical Engineering, Duy Tan University, Da Nang, 550000 Viet Nam

**Keywords:** Environmental chemistry, Applied mathematics

## Abstract

In this study, single-bubble electro-hydrodynamic effects on the two-phase laminar flow of water under electric field stress are investigated using numerical modeling. A 2D axisymmetric model is also developed to study the growth and departure of a single bubble. The phase-field method is applied to track the interphase between liquid and gas. The growth of the attached vapor bubble nucleus to a superheat at 7.0 °C and 8.5 °C are evaluated with 50° and 90° contact angles. The results show that the enhancement of the contact angle changes the velocity and temperature fields around the bubble. It is observed that the growing size and base of the bubble is increased with increasing the wall superheat, but the bubble departure diameter and time are decreased. The electric field results in raising the number of detached bubbles from the superheat at a certain time interval but decreasing the bubbles departure size. Additionally, the formation of stretched bubbles enhances the rate of heat flux and there is a non-linear relationship between the applied voltage and heat flux.

## Introduction

Boiling heat transfer, like nucleate boiling, is one of the efficient types of heat transfer^1^. High heat fluxes can be achieved at low superheats in the nucleate boiling. The thermal function of heat exchangers can be improved by using active and passive heat transfer enhancement techniques^2^. The electric field is one of the active methods to improve heat transfer^3^. In recent years, the use of the electric field in biphasic flows has been increased. The study of the dynamics of electrically charged fluids is known as electrohydrodynamics (EHD). In EHD, the fluid flow equations are evaluated under the influence of an electric field^4^. The electric field can be used to study the hydrodynamics of the bubble and understand the dynamic behavior of bubble deformation and decomposition. It is important for its application in many industries and for the synthesis of materials. Many researchers have studied the effect of the electric field on bubble dynamics^5–10^. Tomar et al. simulated the heat and mass transfer rates in the film’s boiling region and under the influence of a uniform electric field. A combination of the volume of fluid method (VOF) and the level set method (LSM) was used for the simulation. It was found that mass and heat transfer increases with increasing electric field intensity^11^. Gambhire and Thaokar compared the oscillations of the interphase of the two fluids under the influence of a non-uniform electric field for two semiconductor and conductive models. The results showed that controlling interphase instability was better when the electric field was oscillating. It was also shown that oscillating electric fields could control the instabilities at the interphase by increasing the frequency of the applied voltage, for the steady-state system at zero frequency^12^. Ishimoto et al. studied the effect of the non-uniform magnetic field on the behavior of bubbles in magnetic fluid experimentally. It was concluded that it is important to consider the behavior of the bubbles in such cases in many engineering applications such as modern energy conversion systems, but it is difficult to see the bubbles in such currents^13^. The effect of a uniform magnetic field on the air and vapor bubbles in a magnetic field was experimentally investigated. It was found that applying a magnetic field to the bubble flow of the thermal pipes can increase the heat transfer rate and allows it to be controlled. However, it was reported that the observation of the air and vapor bubbles in the liquid under the influence of the magnetic field is difficult^14^. The boiling of a single bubble in a fluid of water under normal and low gravity was examined and it was concluded that the lifetime of the bubble could be adjusted based on the water temperature^15^. As can be seen, many research works have been done experimentally^16–19^ and it has seen a number of limitations and difficulties. Therefore, it will be highly valuable to investigate these systems using modeling and simulation methods^20,21^. For this reason, this research study was conducted using modeling and simulation.

In present work, comprehensive numerical modeling and simulation using the phase-field method were developed to study single-bubble electro-hydrodynamic behavior in the two-phase flow of water under electric field stress. Also, the bubble hydrodynamic was evaluated without electric field stress or gravitational acceleration to see how the electric field can affect the bubble hydrodynamic. The bubble motion and its deformation were studied at operating conditions of laminar flow, incompressible, and unsteady two-phase fluid flow under gravitational and electric fields. Furthermore, a 2D axisymmetric model was developed to investigate the growth and departure of a single bubble. The developed model was verified with experimental data in the absence of an electric field in terms of bubble departure diameter. The results of the current research study can provide valuable insights into relevant experimental research in the field of aerospace research and terrestrial processes.

## Results and Discussion

**Fig. 1:**
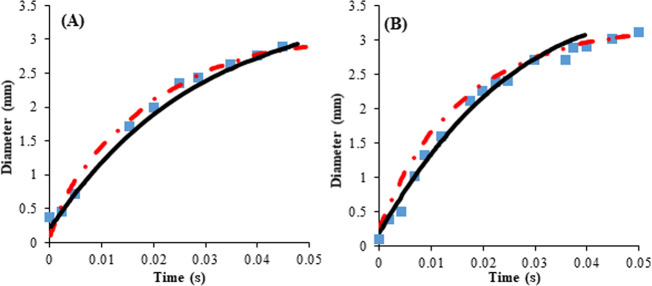
Verification of the developed model with experimental and numerical data. Validation of simulation results of the bubble growth process with published empirical results^22^ and numerical data: (**A**) superheat with temperature and contact angle of 7°C and 50° (**B**) with temperature and contact angle 8.5°C and 50°. Experimental^22^ (), Computational^22^ (), and present work (NOE=9632) ().

To validate the model, the process was simulated without an electric-field and obtained results were compared with the experimental data from the published literature^22^. Then, the effect of the electric-field on the desired parameters was investigated. Modeling results were consistent with the results of experimental research on the evaporation of water in the presence of an electric-field. Figure 1A andB compare the bubble growth time for the experimental data and modeling values when the fluid comes into contact with the superheat with a temperature of 7 °C and 8.5 °C^22^. As can be observed, there is a great agreement between the experimental data and modeling values. Also, the modeling results are more accurate compared to the previous numerical work^22^. From Fig. 1A, the highest error for the bubble growth time was obtained at 11%. In terms of the bubble departure diameter, the maximum error was found to be 2.6%. However, in the superheat with the temperature of 8.5 °C, the error values were slightly higher and those were calculated 16 and 8% for the growth time and the departure diameter, respectively. It can be seen in Fig. 1A and B, the obtained diameter in the simulation is less than the experimental and previous numerical values.

**Fig. 2:**
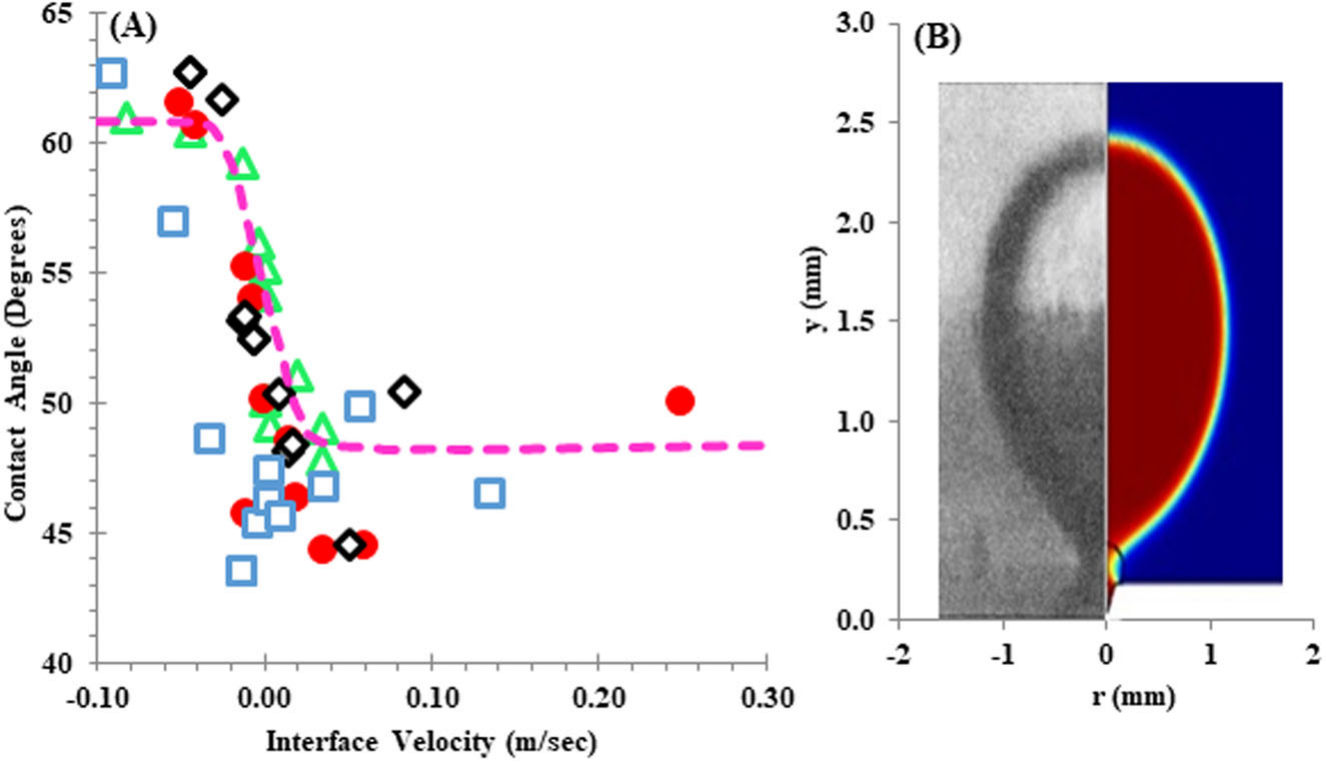
Comparison between the modeling results and experimental data. **A** Comparison of experimental^23^ and modeling results of dynamic contact angle at bubble base in nucleate pool boiling process. **B** Comparison of bubble shape prediction modeling result with experimental for ΔT = 8.5 °C and contact angle = 50°^22^. Present work (T_w_-T_sat_= 7 °C) (), present work (T_w_-T_sat_= 8.5 °C) (), (T_w_-T_sat_= 7 °C)^23^ (), (T_w_-T_sat_= 8.5 °C)^23^ (), and trend line ().

Furthermore, in Fig. 1A and B, mesh size sensitivity analysis was investigated. As can be seen, the change in the meshing from 9632 to 11086 does not affect the results and the error is less than 2%. More information about meshing can be found in Supplementary Section 2 (Supplementary Table 2 and Fig. 2).

The change in the contact angle as a function of the interphase velocity for a single bubble in the current work and the experimental data^23^ from the literature was shown in Fig. 2 (A). From Fig. 2 (A), there is a good agreement between modeling values and the experimental data. Furthermore, when the shape of the bubbles is compared with the empirical results, it can be seen that the shapes of the two bubbles match well for a superheat with temperature and contact angle of 8.5 °C and 50° (Fig. 2 (B)^22^. Also, velocity and temperature fields were given in Supplementary Section 4 (Supplementary Fig. 4).

Electrohydrodynamic force can change the shape of the bubble. This force is the divergence of the Maxwell stress tensor. From Eqs. (1) to (4)^24^, the Maxwell stress tensor consists of two positive and negative terms.

1 $$f + \varepsilon \mu _0\frac{{\partial S}}{{\partial t}} = \nabla T$$

2 $$T_{ij} = \varepsilon _0\varepsilon _r\left( {E_iE_j - \frac{1}{2}\delta _{ij}E^2} \right) + \frac{1}{{\mu _0}}\left( {B_iB_j - \frac{1}{2}\delta _{ij}B^2} \right)$$

3 $$S = \frac{1}{{\mu _0}}\left( {E \times B} \right)$$

4 $$\delta _{ij} = \left\{ {\begin{array}{*{20}{c}} 1 & {i \ne j} \\ 0 & {i = j} \end{array}} \right.$$

**Fig. 3:**
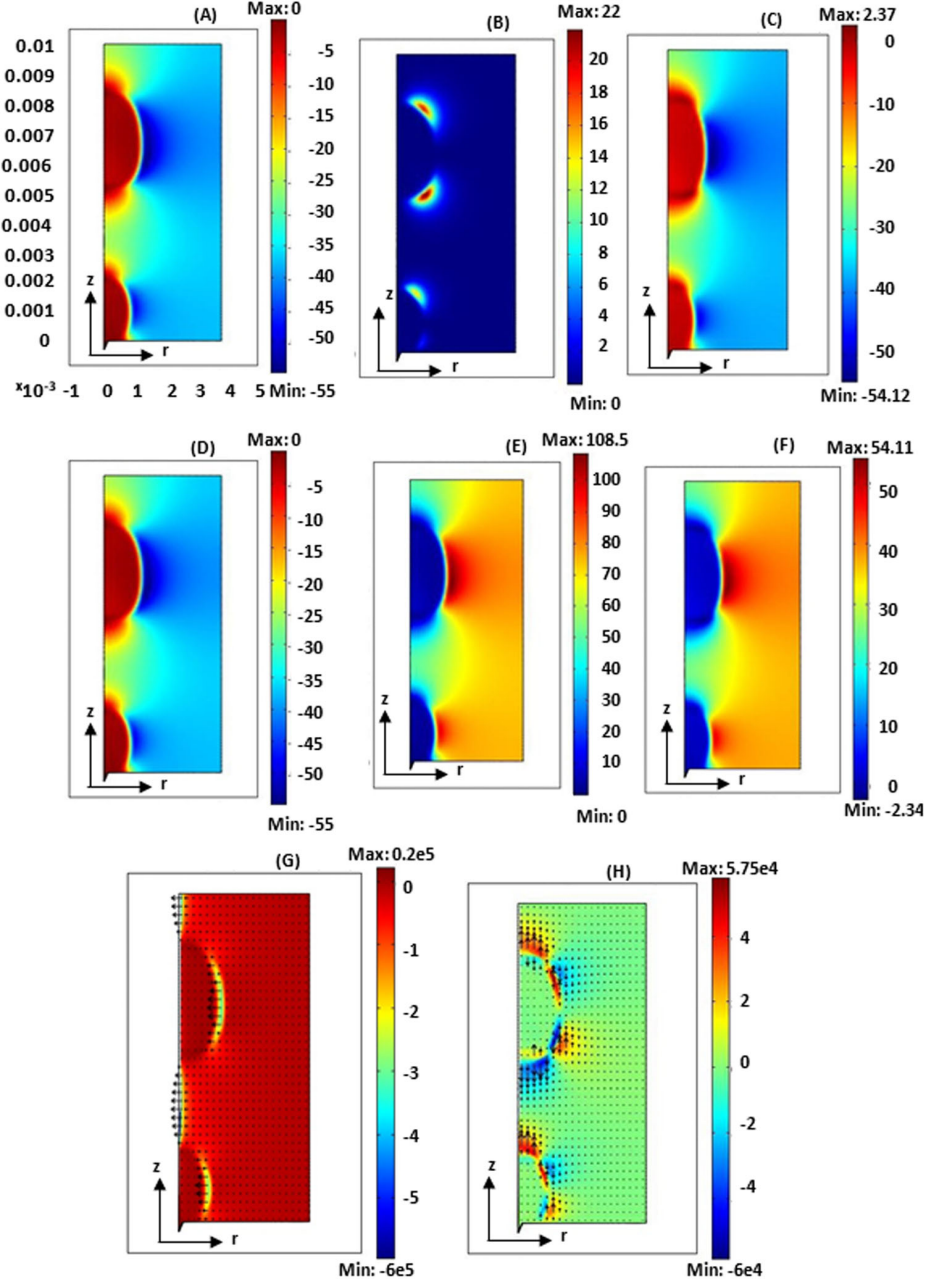
Radial and vertical component of the maxwell tension tensor for superheat 8.5 °C and the contact angle 50° at a voltage of 4000 V. **A**–**C** The radial component of the maxwell tension tensor (**A**) the first term of the radial component of the Maxwell stress tensor (**B**) the second term of the radial component of the maxwell tensile tensor (**C**) the radial component of the Maxwell stress tensor. (**D**–**F**) the vertical component of the maxwell tension (**D**) the first term of the vertical component of the Maxwell stress tensor (**E**) the second term of the vertical component of the maxwell tensile tensor (**F**) the vertical component of the Maxwell stress tensor. (**G**, **H**) electrohydrodynamic force applied to the growing bubble (**G**) radial force (**H**) vertical force. For all (**A** to **H**), the x-axis is the bubble diameter and y-axis is the bubble height and the unit of the x-axis and y-axis is meter. The unit of rainbow colorbars is N (newton). Arrows in **G** and **H** are radial and vertical EHD forces respectively.

In these equations, E is the electric field, B is the magnetic field, S is a closed surface, $\varepsilon$ is the absolute permittivity of space between charges, $\varepsilon _0$ is vacuum permittivity, and $\varepsilon _r$ is the relative permittivity or dielectric constant of the medium. Figure 3 shows the first, the second, and complete terms of the radial component of the Maxwell stress tensor in the sections of (A), (B), and (C), respectively. Negative values indicate the force to the left and positive values indicate the force to the right. As shown in section (A) of Fig. 3A, the force from the first term is applied more to the middle part of the bubble and it causes a transition of the middle part of the bubble from the liquid bulk, to the central axis of the bubble. The second part of the Maxwell stress tensor is zero, except in the small regions of the top and bottom corners of the bubble (shown in Fig. 3B). This force direction is to the right, but it is smaller than the first term. Based on Fig. 3C, there is a force in the middle of the vapor bubble in the left direction but the force direction on the top and bottom of the bubble is upward and downward respectively. Furthermore, the amount of force in the left direction is dominant in the system, therefore, the size of the bubble was decreased with increasing the amount of voltage. From Fig. 3D and F, it was observed that the vertical forces are mostly upward and it can lead to the acceleration of bubble departure from the surface. On the other hand, these forces in the inner corners of the bubble in the upper and lower parts also cause stretching of the bubble shape. The direction of radial and forces were provided in Fig. 3G and H for a clear understanding of effects of different terms in the Maxwell stress tensor.

**Fig. 4:**
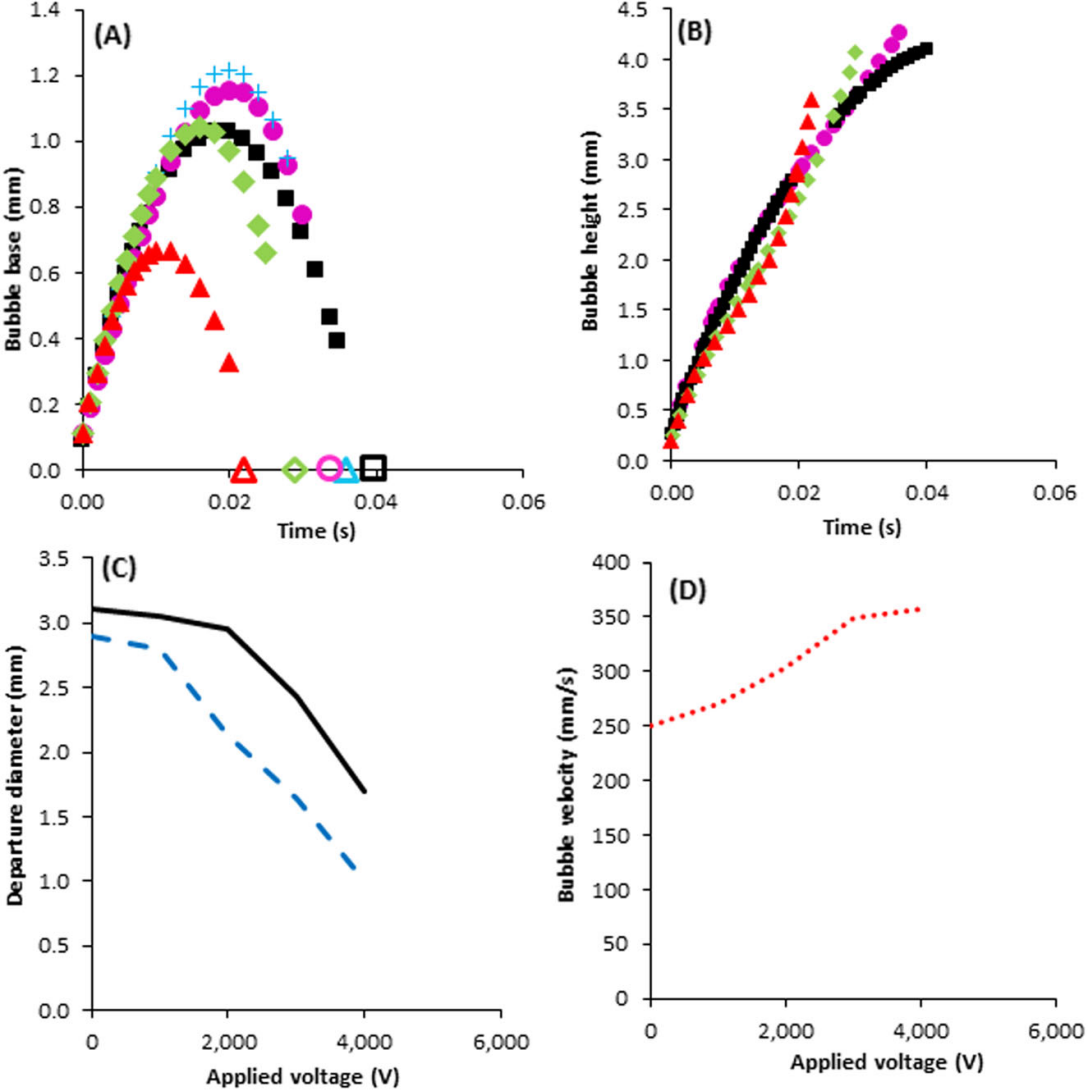
Electrohydrodynamic force effect on the bubble specifications. Electrohydrodynamic force effect on: The bubble base (**A**), height (**B**), diameter (**C**), and departure velocity (**D**). The legends for (**A**) and (**B**) 0V (), 1000V (), 2000 V (), 3000V (), 4000V (), the legends for (**C**) Tw-Tsat = 7°C (), and Tw-Tsat = 8.5°C (), and the legend for (**D**) Tw-Tsat = 8.5°C ().

The effect of electric field on the growing bubble base and height was shown in Fig. 4A and B respectively. In Fig. 4A, the empty symbols on the x-axis are the bubble departure time at different applied voltage. From Fig. 4 (A), the bubble base increased and reached a peak as a function of time, then, it was seen decreasing in the bubble base until its departure from the surface. In the absence of electric field, the maximum bubble base of 1 mm was obtained at time of 0.019 s and the bubble departure from the surface happens at 0.04 s. Applying 4000 V electric field deceased the bubble departure time to 0.022 s. There was an increase in the maximum base from 1 mm to 1.18 mm and 1.20 mm when 1000 V and 2000 V voltages were applied respectively but the amount of time needed for reaching the maximum base was not changed. Moreover, the maximum base was found to be 1 mm when 3000 V voltage was applied but time was decreased from 0.019 s to 0.016 s. Applying 4000 V voltage decreased the maximum value of base and time to 0.64 mm and 0.016 s, respectively. The empty symbols on the horizontal X-axis are the bubble departure time. In addition, the bubble diameter was decreased by applying 3000 V and 4000 V voltage. As it was explained in Fig. 3H, the direction of the vertical forces is upward and it resulted in the decrease in the bubble departure time from the surface (Fig. 4A). Furthermore, the upward and downward vertical forces in the bubble internal corners at the top and bottom of the bubble led to the increase in the growing bubble height when the bubble diameter was the same. There was a bit increase in the bubble height at voltage of 1000 V. The same trend was reported by Zu and Zhang works^25,26^. But, with decreasing the bubble size at a higher voltage value (Fig. 4B), it can be seen a bit decrease in the bubble height at the voltage of 3000 V, and the bubble height was decreased 0.5 mm by applying 4000 V voltage in comparison with the system without any electric field.

The bubble departure diameter can be affected by different forces including buoyancy force, surface tension, and applied electric force. Therefore, electric force can change the bubble size. Electrohydrodynamic force effect on the bubble diameter at the time of departure for the superheat with temperatures of 7 °C and 8.5 °C was presented in Fig. 4C. The bubble departure diameter was decreased from 2.9 mm to 1.0 mm and 3.1 mm to 1.7 mm when the voltage was increased from 0 to 4000 V for the superheat with temperatures of 7 °C and 8.5 °C. As can be seen, the higher electric force was required to significantly decrease the departure diameter of the bubble. This means that a larger inward force is needed to overcome the expansion pressure and the volumetric growth of the bubble which enters from the vapor phase to the liquid phase. The decrease in the bubble diameter could be attributed to the existence of radial electrohydrodynamic force in the middle of the bubble (Fig. 3G).

Figure 4D shows the change in the bubble velocity as a function of the applied voltage. In the present study, the bubble velocity was measured at 1 cm from the bottom of the container. It was observed that the bubble velocity was increased from 250 mm/s to about 360 mm/s when the applied voltage was increased from 0 to 4000 V. This result is consistent with the experimental work of Kweon and Kim^27^.

After the bubble growth and its departure from the hot surface, the cooler saturated liquid fills the position of the separated bubble from the hot surface, then, a certain time is required for heating of liquid and nucleation of the bubble. The growth time (t_g_) and waiting time (t_w_) can be used for the calculation of the average bubble frequency (*f*) for a bubble using the following equation:^27^

5 $$f = \frac{1}{{t_w + t_g}}$$

**Fig. 5:**
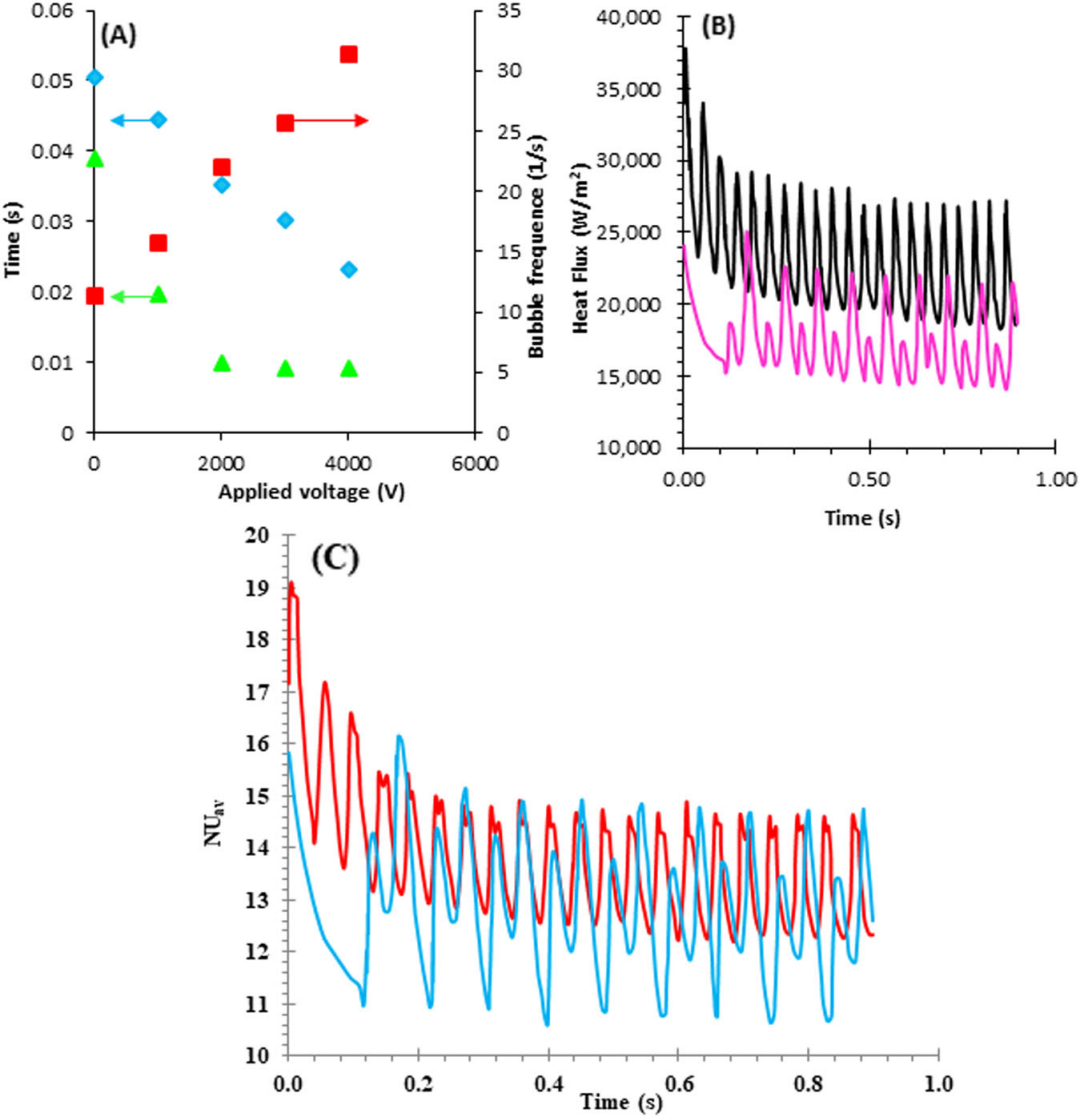
Effect of applied voltage on the bubble behavior in the system and change in heat flux and Nusselt number with time. (**A**)The change in growth time, waiting time, and average frequency as a function of applied voltage. (**B**) Integration of heat flux passing through the wall. (**C**) The trend of changes in the average Russell number on the wall versus time. Wating time (), growth time (), Frequency (), heat flux (Tw-Tsat = 7 °C) (), heat flux (Tw-Tsat = 8.5 °C) (), Nuav (Tw-Tsat = 7 °C) (), Nuav (Tw-Tsat = 8.5 °C) ().

The change in growth time, waiting time, and the average bubble frequency as a function of the applied voltage is shown in Fig. 5A. As can be seen, the growth time is always higher than the waiting time for all voltages and the case without electrical force. Kweon et al.^27^ reported the same behavior for water but based on the fluid type it is possible to have a waiting time higher than the growth time. There was a decrease in the growth and waiting times with increasing applied voltage and subsequently the amount of the average frequency was increased with the enhancement of applied voltage. It is clear based on Eq. (5) and the same trend was reported by Kweon et al.^27^ Moreover, it was observed that the waiting time was not changed when the applied voltage was increased from 2000 V to 4000 V. It is because there is a direct relationship between waiting time and thermal boundary layer. The voltage higher than 2000 V was not able to change the thermal boundary layer. Therefore, it was remained constant between 2000 V and 4000 V.

The fluid velocity is zero on the surface of the hot wall, therefore, there are two mechanisms for the heat flux through the wall include conductive heat flux and the heat flux due to phase change from liquid to vapor and it is maximum at the triple point on the wall. The heat flux can change with the change in the vapor nucleation, growth, and its departure from the surface. The change in heat flux as a function of time (900 ms) for the superheat with the temperature of 7 °C (9^th^ bubbles) and 8.5 °C (20^th^ bubbles) was shown in Fig. 5B. The vapor phase thermal conductivity coefficient is too lower than the liquid phase. Therefore, thermal conductivity was too much lower where the vapor was formed. The heat flux was decreased during bubble growing and increasing of bubble base, but it was then increased with decreasing bubble base and beginning of the bubble departure step. It can be seen a slight decrease in heat flux when the process goes from one bubble to the next one. In addition, it was observed that there are small peaks between large peaks when the superheat temperature is 7.5 °C. It is because the waiting time is longer for this case and vapor nucleation occurs after a certain time interval when a bubble departure from the surface.

Using dimensionless number is more appropriate to compare different samples or parameters. Nusselt dimensionless number is defined as follows:

6 $$Nu = \frac{{l_0q}}{{k{\Delta} T_{sup}}}$$

where *q* and *k* are heat flux and thermal conductive coefficient. The difference between Nusselt numbers at different applying voltage is lower than the difference between heat flux. It is because ΔT_sup_ is the denominator in the fraction (Fig. 5C). As it can be seen, the distance between the Nusselt diagrams is less than the distance between the graphs of the two superheated fluxes (Fig. 5B), because although the heat flux is higher at 8.5 °C the lower ΔT_sup_ at 7 °C reduces the difference between the two Nusselt number values.

**Fig. 6:**
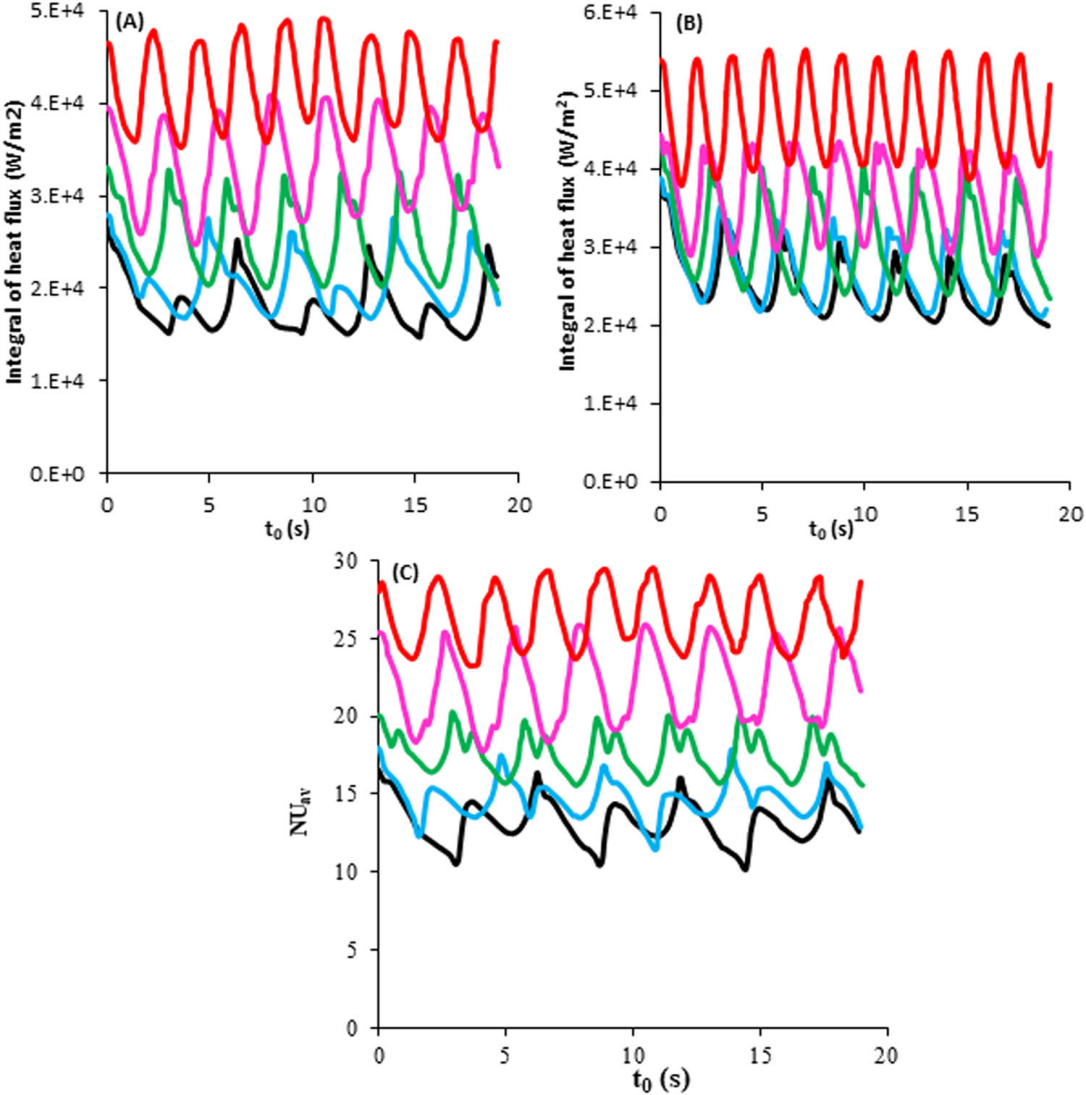
Heat flux and Nusselt number passing through the superheat at different operating conditions. Integration of heat flux at different voltage passing through the superheat with contact angle of 50° and temperature of (**A**) 7°C and (**B**) 8.5°C. (**C**) Nusselt number at different voltage passing through the superheat with the temperature of 7°C and contact angle of 50°. 0V (), 1000V (), 2000 V (), 3000V (), and 4000V ().

Figure 6A and B show the effects of electric field and time on the heat flux passing through hot surfaces with temperatures of 7 °C and 8.5 °C during a certain time, respectively. The contact angle was 50 degrees. It was observed that increasing applying voltage increased the heat transfer rate and the number of bubbles which departure the surface. For example, the number of bubbles was increased from 3 to 9 when the applied voltage was enhanced from 0 to 4000 V for the case with a superheat temperature of 7 °C. As it was explained, applying voltage decreases growth and waiting times. Therefore, the heat transfer rate through superheats increases with increasing of the average bubble frequency and bubble departure. Also, Electrohydrodynamic force can change streamlines close to the superheat and bubble. All these changes can affect the temperature field in the system and subsequently improve the heat transfer rate. In addition, it was found that increasing applying voltage was decreased the thermal boundary layer and this is also increased the heat flux rate.

From the comparison of Fig. 6A (7.0 °C) and 6B (8.5 °C), it was found that increase in the heat flux is higher for the system without any electric field as the heat flux increased from 26000 W/m^2^ to 38000 W/m^2^ while when 4000 V voltage was applied there was an only 7000 W/m^2^ increase in the heat flux (from 48000 W/m^2^ to 55000 W/m^2^). Therefore, the impact of superheat temperature was higher in the absence of an electric field.

The Nusselt number as a function of time and voltage is shown in Fig. 6C. The maximum Nusselt number was increased from 16 to about 29 with increasing applying voltage from 0 to 4000 V.

**Fig. 7:**
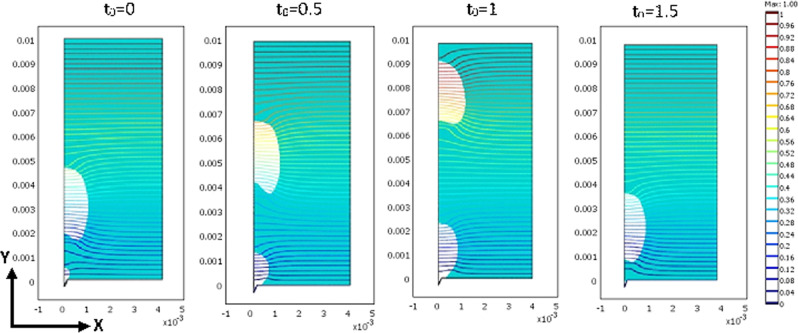
Distribution of dimensionless electric potential for the superheat with temperature of 8.5 °C and contact angle of 50°. The unit of x-axis and y-axis is meter.

The distribution of electrical potential depends on the shape and the position of the vapor bubbles in the computational domain. Figure 7 shows the distribution of dimensionless electrical potential in the computational domain. Electrical permeability is different for the liquid and gas phases which is clearly shown in Fig. 7. The difference in the electrical permeability of liquid and vapor phases could be attributed to the different electrical volume force gradian in each phase. In fact, the bubble acts as a barrier for the passing of the magnetic field due to its lower magnetic permeability. On the other hand, the fluid flow tends to remove this barrier for the passing of the electrical field. The applied force is only effective on the interphase of two phases. Therefore, it can be called magnetic surface tension. The electrical field is a straight line for one phase. But, it can be seen a change in the electrical field, when it penetrates from the bubble to the liquid phase.

Further results and discussion are provided in Supplementary Section 4 (Supplementary Figs. 4 to 8)

## Methods

### Governing equations

The system geometry and fluid properties were provided in Supplementary Section 1 (Supplementary Fig. 1 and Table 1). The main equations for the model building are conservation equations for the mass and momentum in the incompressible state. The velocity and pressure fields for the liquid phase were modeled by the Navier-Stokes equations, according to Eqs. (7) and (8)^28^.

7 $$\rho _l\frac{{\partial u_l}}{{\partial t}} + \rho _l\left( {u_l.\nabla } \right)u_l = - \nabla .\left( {\rho _l.I} \right) + \nabla .\left[ {\eta _{\mathrm{l}}.\left( {\nabla {\mathrm{u}}_{\mathrm{l}} + \left( {\nabla {\mathrm{u}}_{\mathrm{l}}} \right)^{\mathrm{T}}} \right)} \right] + \rho _lg$$

8 $$\nabla .u_l = 0$$

where $\rho _l$, $u_l$, ${\eta _{\mathrm{l}}}$ are the fluid density, fluid velocity, and fluid dynamic viscosity, respectively, and l subtitle represents the liquid phase. For the vapor phase, a weak form of Navier Stokes equations was used, according to Eqs. (9) and (10)^28^:

9 $$\rho _v\frac{{\partial u_v}}{{\partial t}} + \rho _v\left( {u_v.\nabla } \right)u_v = - \nabla .\left[ { - \rho _v.I + \eta _{\mathrm{l}}.\left( {\nabla {\mathrm{u}}_{\mathrm{l}} + \left( {\nabla {\mathrm{u}}_{\mathrm{l}}} \right)^{\mathrm{T}}} \right) - \frac{2}{3}\eta \left( {\nabla .u} \right)I} \right] + \rho _vg$$

10 $$\frac{{\partial \rho _v}}{{\partial t}} + \nabla \left( {\rho _vu_v} \right) = 0$$

where u and η are the fluid bulk velocity and the dynamic viscosity of the fluid bulk, respectively, $u_v$ is the fluid velocity of the vapor phase, and $\rho _v$ is the fluid density of the vapor phase. The energy equation was solved only for the vapor phase based on Eq. (11)^29^:

11 $$\rho _v.C_p\frac{{\partial T_v}}{{\partial t}} + \rho _v.C_p\left( {{\mathrm{u}}_{\mathrm{v}}.\nabla } \right){\mathrm{T}}_{\mathrm{v}} = - \nabla .k_v\nabla T_v$$

where $C_p$ is the heat capacity of the vapor phase and $k_v$ is the heat transfer coefficient of the vapor phase. The interphase temperature of the vapor-liquid was considered to be equal to the saturation temperature, therefore, the heat transfer equation was solved only for the vapor phase. The Poisson equation (Eq. (12)) was also solved as the electric field equation.

12 $$\nabla .{\mathrm{E}} = \nabla .\left( { - \nabla {\mathrm{V}}} \right) = - \nabla ^2V = \frac{{\rho _f}}{\varepsilon }$$

This function is Poisson’s equation for the dielectric materials^30^.

### Domain equations

In the phase-field method, instead of direct tracking of the interphase between the two phases, the intermediate layer is obtained by the phase-field variable. It is assumed that the state of the system is described at any time by the phase-field variable $\phi$, which is a function of the position vector. The Cahn–Hilliard equation is used for describing the dynamics of the interphase in two-phase flow as follows (shown in Eq. (13))^31^:

13 $$\frac{{\partial \phi }}{{\partial t}} + u \cdot \nabla \phi = \nabla \cdot {\upgamma}\nabla {\mathrm{G}}$$

where G (Pa) is the chemical potential and *γ* (m^3^.s/kg) is the mobility parameter. The free energy is a function of the dimensionless parameter of the phase-field is presented based on Eq. (14):

14 $$F\left( {\emptyset ,\nabla \emptyset ,T} \right) = {\int} {\left( {\frac{1}{2}\varepsilon ^2\left| {\nabla \emptyset } \right|^2 + {\mathrm{f}}\left( {\emptyset ,{\mathrm{T}}} \right)} \right)} {\mathrm{dV}} = {\int} {{\mathrm{f}}_{{\mathrm{tot}}}{\mathrm{dV}}}$$

where ε is the value of the interfacial thickness and f_tot_ (J/m^3^) is the total free energy density of the system. The free energy density relating to a mixture of isotherm consisting of two insoluble fluids is a summation of mixing energy and elastic energy. The mixing energy was estimated by the Ginzburg–Landau Eq. (15):^31^

15 $$f_{mix}\left( {\emptyset ,\nabla \emptyset } \right) = \frac{1}{2}\lambda \left| {\nabla \emptyset } \right|^2 + \frac{{\uplambda }}{{4{\upvarepsilon}^2}}\left( {\emptyset^2 - 1} \right)^2$$

The dimensionless parameter of the phase-field $\emptyset$ is determined in such a way that the volume fraction of the fluid components is $(1 + \emptyset )/2$ and $(1 - \emptyset )/2$. The symbol $\lambda \left( N \right)$ is the mixing energy density. This parameter is related to Eq. (13 in SIF file), the surface tension coefficient, $\sigma \left( {{\mathrm{N}}/{\mathrm{m}}} \right)$, and the thickness of the interphase. The degree of mobility determines the time scale of the diffusion of the Cahn–Hilliard, and it should be large enough to have a constant interfacial thickness, and also it should be small enough to avoid the over-damping of the convective terms. The degree of mobility is related to the thickness of the interphase using the mobility tuning parameter $\chi \left( {m.s/kg} \right)$ which is determined according to $\gamma = \chi \varepsilon ^2$. The value of the chemical potential is obtained by the following Eq. (16)^32^:

16 $$G = \frac{{\delta {\int} {{\mathrm{f}}_{{\mathrm{mix}}}{\mathrm{d}}{\Omega} } }}{{\delta \emptyset }} = \lambda \left[ { - \nabla ^2\emptyset + \frac{{\emptyset \left( {\emptyset^{2} - 1} \right)}}{{\varepsilon ^2}}} \right]$$

Hence, the right side of Eq. (12) is for the minimization of the total free energy using the residence time, which this time was controlled by motion parameter $\gamma \left( {m^3{\mathrm{s}}/{\mathrm{kg}}} \right)$. The Cahn–Hilliard function forces the parameter $\emptyset$, except in very narrow regions of the fluid interphase, to accept two values of 1 or −1 and by breaking the fourth-order equation into the following two second-order equations Eqs. (17) and (18):

17 $$\frac{{\partial \emptyset }}{{\partial t}} + {\mathrm{u}}.\nabla \emptyset = \nabla .\frac{{\gamma \lambda }}{{\varepsilon ^2}}\nabla \psi$$

18 $$\psi = - \nabla .\varepsilon ^2\nabla \emptyset + \left( {\emptyset^2 - 1} \right)\emptyset$$

The term of free energy can sometimes include other sources. These sources can be considered by modifying the Eq. (18) to the following equation:

19 $$\psi = - \nabla .\varepsilon ^2\nabla \emptyset + \left( {\emptyset^2 - 1} \right)\emptyset + \left( {\frac{{\varepsilon ^2}}{\lambda }} \right)\frac{{\partial f_{ext}}}{{\partial \emptyset }}$$

where $f_{ext}$ is the determined free energy by the user (J/m^3^). The external free energy is mostly zero^31^.

### Phase-field method in solution of two-phase problem

To solve the two-phase problem of the boiling process with the phase-field method (PFM), firstly, the boundary and initial conditions and fluid properties in both phases and the motion parameter of the phase-field model must be determined. Then, the variables of the laminar flow model of this method include the dimensionless parameter of phase ($\emptyset$), and the auxiliary variable $\psi$ in the computational domain must be initialized. In the next step, the transient analysis type is selected so that the main solution of the model is performed with all variables of flow equation including (p) pressure, the dimensionless variable of phase ($\emptyset$), auxiliary variable (ψ), (u) and ($\nu$) velocities, energy equation including temperature variable (T) and electrostatic equation including variable of potential difference (V). In this step, a number of terms is added to the equations as a source to consider the effect of phase change. The following terms are added to the equations of momentum, energy, and phase, Eqs. (20), (21) and (22) respectively:

20 $$\nabla .u = {\dot{\mathrm m}}\delta \left( {\frac{1}{{\rho _{\mathrm{l}}}} - \frac{1}{{\rho _{\upnu}}}} \right)$$

21 $$\rho {\mathrm{C}}_{\mathrm{p}}\frac{{\partial {\mathrm{T}}}}{{\partial {\mathrm{t}}}} + \rho {\mathrm{C}}_{\mathrm{p}}\left( {{\mathrm{u}}.\nabla } \right){\mathrm{T}} = - \nabla .k\nabla T - \frac{{\dot m\delta {\Delta} H_{\nu l}}}{{M_w}}$$

22 $$\frac{{\partial \emptyset }}{{\partial {\mathrm{t}}}} + {\mathrm{u}}.\nabla \emptyset - {\dot{\mathrm m}}\delta \left( {\frac{{{\mathrm{V}}_{{\mathrm{f}}\nu }}}{{\rho _{\mathrm{l}}}} - \frac{{{\mathrm{V}}_{{\mathrm{fl}}}}}{{\rho _\nu }}} \right) = \nabla .\frac{{\gamma \lambda }}{{\varepsilon ^2}}\nabla \psi$$

where ${\updelta}$ is the length of the interphase between two phases based on the Eq. (23):

23 $$\delta = 6{\mathrm{V}}_{\mathrm{f}}\left( {1 - {\mathrm{V}}_{\mathrm{f}}} \right)\frac{{\left| {\nabla \emptyset } \right|}}{2}$$

In this way, the variables of the model are obtained over time, and then the physical and thermal properties of the fluid are calculated. The volume fraction amount of vapor and liquid is calculated using the phase-field variable given in Eq. (24):

24 $${\mathrm{V}}_{\mathrm{f}} = {\mathrm{min}}\left( {{\mathrm{max}}\left( {\left[ {\frac{{1 + \emptyset }}{2}} \right],0} \right),1} \right)$$

Min and max operators were used to measure the volume fraction between the lower and upper limits of 0 and 1. Thus, mixed properties such as specific heat density, thermal conductivity coefficient, and electrical permeability are obtained using the volumetric component and the following Eq. (25):

25 $${\mathrm{A}} = \left( {{\mathrm{A}}_{\mathrm{l}} - {\mathrm{A}}_{\mathrm{g}}} \right){\mathrm{V}}_{{\mathrm{f}},{\mathrm{l}}} + {\mathrm{A}}_{\mathrm{g}}$$

where A can be any of the properties mentioned, and the subtitles l and g are related to the liquid and gas phases, respectively.

Surface tension force was applied as a volume force, shown in Eq. (26):

26 $${\mathrm{F}}_{{\mathrm{st}}} = {\mathrm{G}}.\nabla \emptyset$$

where G is obtained by Eq. (16). The value of mean curvature is also obtained by Eq. (27)^33^:

27 $${\mathrm{k}} = 2\left( {1 + \emptyset } \right)(1 - \emptyset )\frac{{\mathrm{G}}}{\sigma }$$

### Boundary conditions

The boundary conditions for the boiling model are complex. The interphase velocity of the two phases is not necessarily equal to the velocity of the liquid phase or the velocity of the vapor phase, shown in Eq. (28):

28 $$u_{int} = u_l - \frac{{\dot m}}{{\rho _l}}n$$

In Eq. (28), n is the unit normal vector and its direction is from the liquid phase to the vapor phase. Also, $\dot m$ is the rate of vapor production. The boundary conditions of the vapor phase in the interphase were considered as follows (based on Eq. (29)):

29 $$n.\rho _v.u_v = \dot m\left( {1 - \frac{{\rho _v}}{{\rho _l}}} \right) + \left( {n.\rho _v.u_l} \right)$$

If no phase change occurs, $u_v = u_l = u_{int}$, and the velocity of the solution when passing through an interphase of the two phases is continuous. In addition, if the density of the vapor and liquid phases is equal, the first term on the right side of the Eq. (29) will be zero and the velocity of the two phases is equal. When the phase change occurs and the density of the two phases is not equal, the first term on the right side of the equation has a value and a stream flows into the interphase of the two phases. The second term on the right gives the velocity in the outward direction of the interphase, therefore discontinuity is created in the field of velocity passing through the interphase. The velocity of the liquid in the outer direction of the vapor phase is greater than the vapor phase. The mass flux that leaves the interphase can be written as follows:^34^

30 $$\dot m = - \left( {\frac{{M_w}}{{{\Delta} H_{l.v}}}} \right)\left( {n.k_l.\nabla T_v} \right)$$

where $M_w$ is the molecular mass of the vapor and ${\Delta} H_{l.v}$ is the vapor enthalpy. This equation is approximated by ignoring the kinetic energy and work done by viscous forces. In the interphase of the two phases, three forces affect the liquid phase, so the boundary conditions resulting from the balance of these forces for the liquid phase can be expressed as follows:^35^

31 $$n.\left[ { - p_lI + \eta _l\left( {\nabla u_l + \left( {\nabla u_l} \right)^T} \right)} \right] = \dot m\left( {u_l - u_v} \right) + n.\left[ { - p_vI + \eta _v\left( {\nabla u_v + \left( {\nabla u_v} \right)^T} \right)} \right] + F_{st} + F$$

The first term on the right, the equation is related to the force that separates the vapor from the liquid. This force is always negligible. The second term is the sum of compressive and viscosity forces which is applied to the liquid phase from the vapor phase. The mass flux that leaves the liquid surface increases the vapor phase pressure. This pressure exerts a force on the liquid surface and the vapor region expands. The third term is the force due to surface tension. Surface tensile forces cause pressure discontinuities in the passage of the interphase. The last term in Eq. (31) is the determined volume force by the user, which can be different in various problems. In the present work, F is the electrostatic force that is the divergence of Maxwell stress tensor. In the energy equation, the temperature in the interphase is considered equal to the saturation temperature, which is a function of the pressure, $T = T_{sat}\left( p \right)$.

In the electric field equation, in the interphase, the continuity equation of the electric field is valid, $n.\left( {D_1 - D_2} \right) = 0$.

### Determine of initialization

The phase-field function should have an initial value and it should change from 1 to −1 along with the interphase of two phases. In initialization, the convection terms are not considered and the two equations of partial differential (Eq. (17)) and (Eq. (18)) are solved. Time in the initialization should be determined according to the general characteristics of the problem. Unlike the level set method (LSM), it may be necessary that the time in initialization be much longer than the original simulation time^33^. By selecting different ε values, the phase-field parameter changes on the interphase so the choice of the controlled parameter of the interphase thickness in the initialization and subsequently in the main solution is very important. Supplementary Section 3 (Supplementary Fig. 3) provides more information in this regard. The general value for this parameter is equal to half of the maximum meshing used for the problem- solving. But, if different parts of the domain have a large difference in mesh size, the mesh size in the interphase section should be the basis for quantifying the thickness controller parameter. The phase-field method, like the level set method, does not require any expression for the normal unit of the interphase or the smoothed delta function. The determining variable in this method is the value of chemical potential that is rewritten based on the dependent variable $\psi$, according to Eq. (32)^33^.

32 $${\mathrm{G}} = \frac{{\lambda \psi }}{{\varepsilon ^2}}$$

**Fig. 8:**
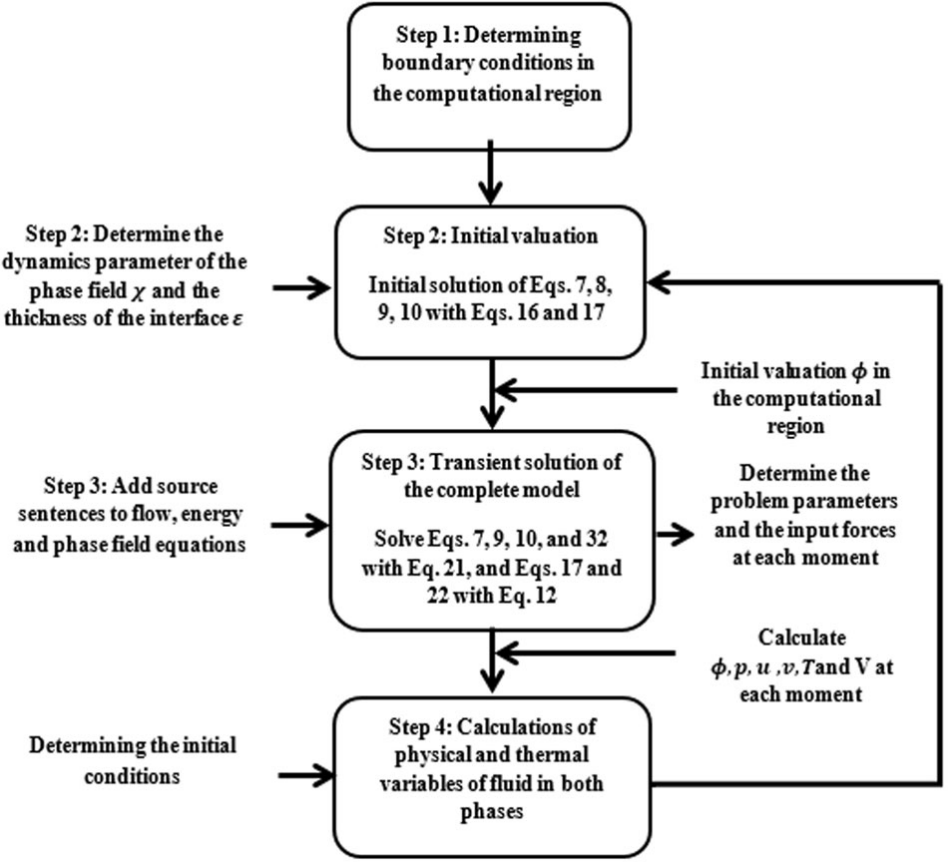
Problem-solving flowchart. The flowchart provides different steps including determination of boundary conditions, initial valuation, transient solution of the complete model, and calculation of fluid properties used in the developed model.

The flowchart for the developed model and the simulation was provided in Fig. 8.

### Bubble dynamics in a gravitational field

Different forces including viscous, electric field, surface tension, inertial, and gravity forces involved in the bubble motion and deformation. The momentum conservation equations in the electrical and gravitational field can be written as follows:^36^

33 $$\frac{{\partial \left( {\rho u} \right)}}{{\partial t}} + \nabla .\left( {\rho uu} \right) = - \nabla {\mathrm{p}} + \nabla .\left[ {\mu \left( {\nabla u + \nabla u^T} \right)} \right] + \rho g + F_\sigma + F_e$$

where u defines the velocity (m s^−1^), the symbols ρ, p, t, g, and μ are the density (kg m^−3^), pressure (Pa), time (s), gravitational acceleration (m s^−2^), and the dynamic viscosity (Pa.s) respectively. Also, F_σ_ and F_e_ denote the surface tension and electric field force. It should be noted that the gravitational acceleration is only considered in the system, when there is no electrostatic force in the system.

Grace^37^ described the single bubble motion and deformation and motion using four dimensionless numbers consisting of the density ratio (λ_ρ_), Morton number (M), viscosity ratio (λ_μ_), and Eotvos number (Eo) in the gravitational field:

35 $$\lambda _\rho = \frac{{\rho _l}}{{\rho _g}}$$

36 $$M = \frac{{\left| g \right|\mu _l^4}}{{\rho _l\sigma ^3}}$$

37 $$\lambda _\mu = \frac{{\mu _l}}{{\mu _g}}$$

38 $$Eo = \frac{{4\left| g \right|R^2\left( {\rho _l - \rho _g} \right)}}{\sigma }$$

These dimension numbers provide the relative importance between two forces. For example, the Morton number is related to surface tension and viscous force^36^. In the current study, the bubble dynamics were investigated in the combined gravitational. The bubble behaviors in the gravitational field were characterized by the following dimensionless parameters.

42 $$Bo_e = \frac{{\varepsilon _0\varepsilon _l\left| {E_0} \right|^2R}}{\sigma }$$

43 $$\lambda _\varepsilon = \frac{{\varepsilon _l}}{{\varepsilon _g}}$$

**Fig. 9:**
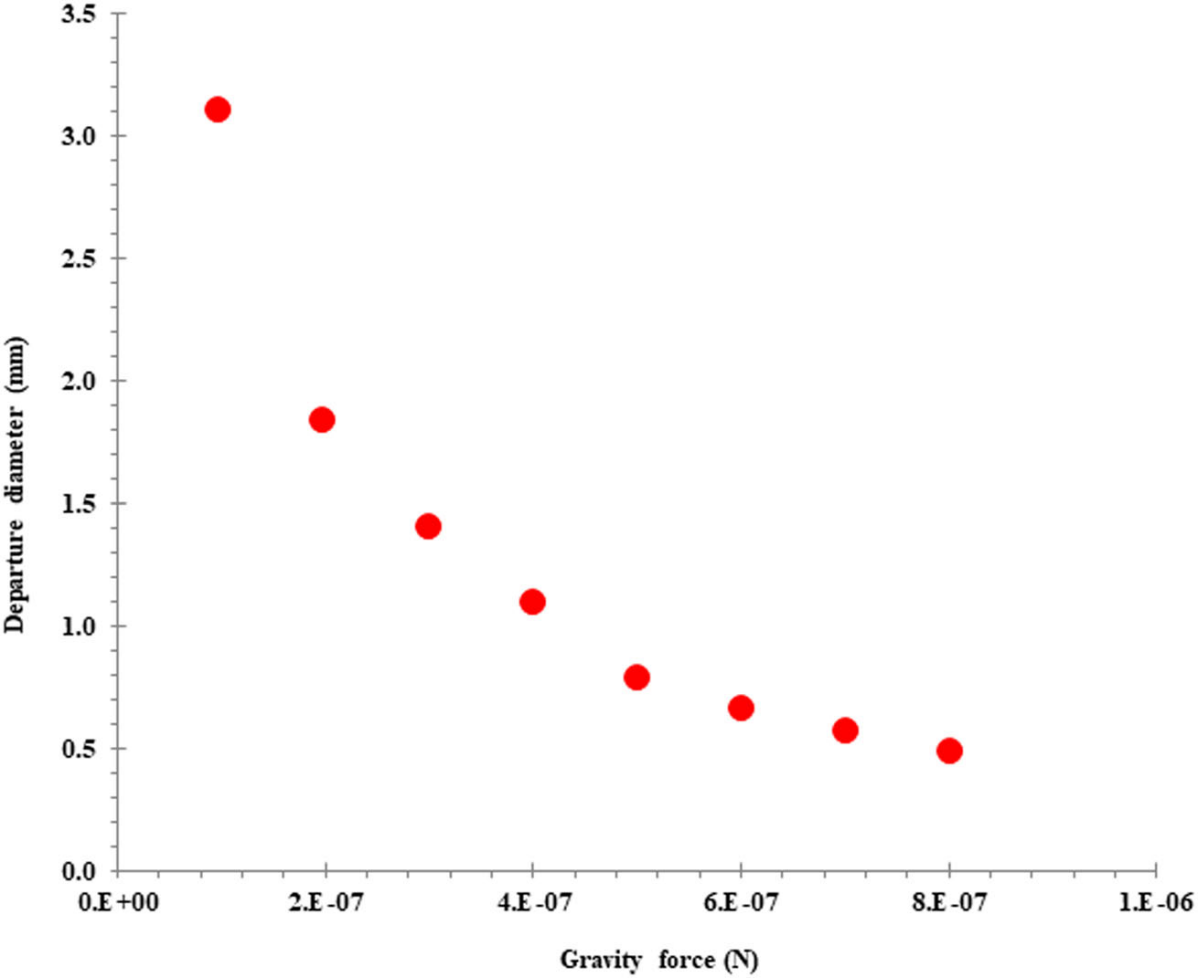
Effect of gravity force on the bubble departure diameter. The change in bubble diameter with increasing the gravity force from 1e^–7^ N to 8e^–7^ N.

The bubble deformation and motion were studied using the Bond number (Bo_e_), Morton number (M), electric and permittivity ratio (λ_ε_), and Eotvos number (Eo) dimensionless numbers. The bubble diameter as a function of gravity force is given in Fig. 9. It was observed that the increasing gravity force led to the reduction of the bubble diameter. It could be attributed to the enhancement of the fluid velocity with increasing gravity and buoyancy force and subsequently less time for the bubble to grow.

In the present work, a high voltage electric field in a fluid medium was used. It was adjusted based on the Earth’s gravitational field. Gravitational force is weak. But, coulomb electric force between a proton and an electron is about 10^39^ times higher than gravitational forces between them^38^.

### Reporting Summary

Further information on research design is available in the Nature Research Reporting Summary linked to this article.

## Supplementary information

Supplemental material

Reporting Summary Checklist

## Data Availability

All data generated or analyzed during this study are included in this published article (and its supplementary information files).
