# Supplementary material for: Single-bubble EHD behavior into water two-phase flow under electric-field stress and gravitational acceleration using PFM
Source: NPJ Microgravity. 2021 Feb 18;7:6. doi: 10.1038/s41526-021-00134-y (PMC7893078; doi:10.1038/s41526-021-00134-y)
Supplement: Supplementary file 1 — Supplemental material [file 41526_2021_134_MOESM1_ESM.pdf]

Supplementary Materials for

Single-bubble EHD behavior into water two-phase flow under electric-field stress and gravitational acceleration using PFM

Maryam Aliakbary Mianmahale[1], Arjomand Mehrabani-Zeinabad[1], Masoud Habibi Zare[1], Mahdi Ghadiri[2,3,*]

[1]Isfahan University of Technology, Department of Chemical Engineering, 84156-83111 Isfahan, Iran

[2]Institute of Research and Development, Duy Tan University, Da Nang 550000, Viet Nam

[3]The Faculty of Environment and Chemical Engineering, Duy Tan University, Da Nang 550000, Viet Nam

[*]Corresponding author; E-mail: mahdighadiri@duytan.edu.vn

Supplementary Section 1: System geometry and liquid and vapor properties

Investigation and understanding of different parameters effect on the boiling process is highly important. A single bubble behavior was studied due to the complexity of boiling process. Initial nucleation of vapor bubble was considered at the centre of a heating cylindrical container from the bottom. Also, the bottom and top of the cylindrical container were round plate electrodes which there is potential difference (ΔV) between two electrodes. It was assumed that the bottom plate has temperature of T_{sup}. The geometry of cylindrical container was shown in Supplementary Fig. 1. The developed equations can be solved for a quarter of the cylinder due to the existing symmetries in the system. The diameter of cylinder was considered 8 mm. The height of the computational domain was determined in such way that the bubble does not leave the domain before departure from the surface. The radius of the vapor initial nucleation was assumed 0.1 dimensionless length and the radius of the hole was considered 150 μm based on the equation reported by Hsu [1].

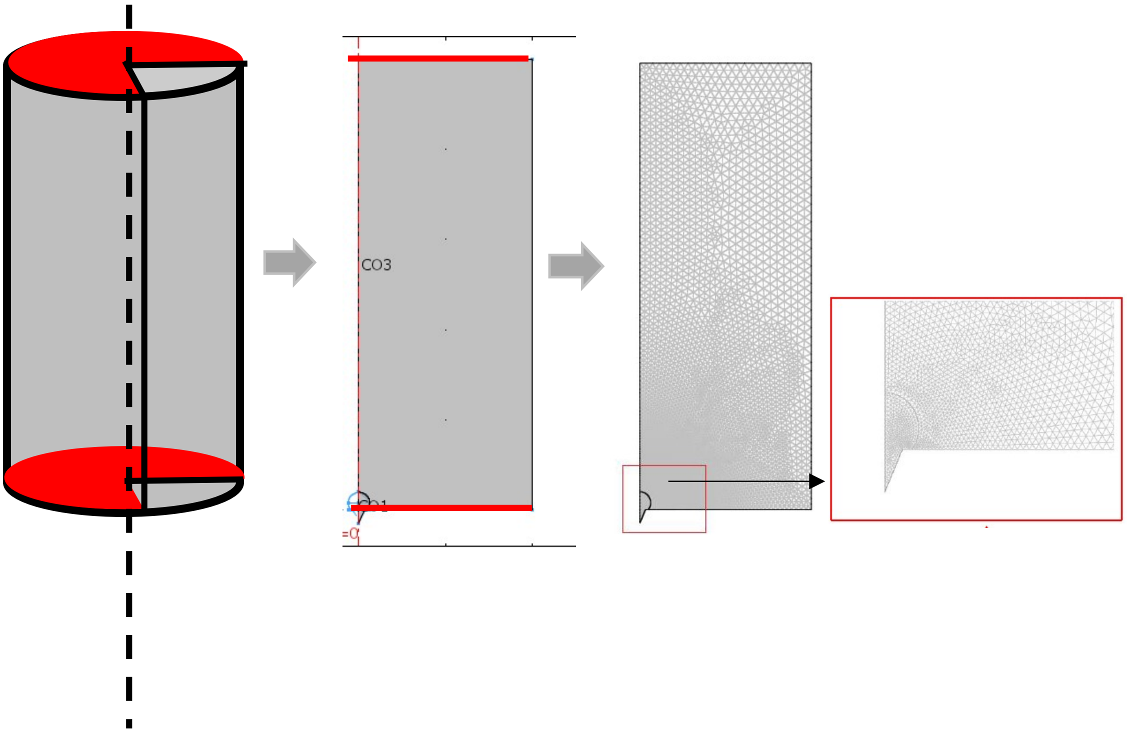

Supplementary Fig. 1 Geometry of computational domain.

The dimensionless characteristics for comparing numerical and experimental works are defined as follows:

$$I_0 = \sqrt{\frac{\sigma}{g(\rho_l - \rho_v)}} \tag{1}$$

$$u_0 = \sqrt{g.l_0} \tag{2}$$

$$t_0 = \frac{l_0}{u_0} \tag{3}$$

$$T^* = \frac{T - T_{sat}}{T_w - T_{sat}} \tag{4}$$

where l_0, u_0, t_0 and T^* are the characteristic length, velocity, characteristic time, and dimensionless temperature respectively. The properties of water and its vapor used in the simulation were provided in Supplementary Table 1.

Supplementary Table 1 Properties of used liquid and vapor in the simulation

Properties	Unit	Liquid	Vapor
**Density**	$Kg.m^3$	958	Eq.38
**Special Heat at Constant Pressure**	Kj/Kg.K	4.212	2.02
**Thermal Conductivity**	W/m.K	0.68	Eq.37
**Viscosity**	m^2/s	$2.85*10^{-4}$	$1.2*10^{-5}$
**Enthalpy of Vaporization**	Kj/Kg	2257	-
**Saturated Temperature**	K	-	373.15
**Volume Expansion Coefficient**	K^{-1}	$7.5*10^{-4}$	-
**Surface Tension**	m^2/s	Eq.7	
**Relative Electrical Permeability**	-	54.5	1.006

Density, thermal conductivity of the vapor, and also surface tension depend on the temperature, so for these properties, temperature-dependent functions were used in the software. The three functions of density, thermal conductivity and surface tension are as follows:

$$k_g = 8.3154 \times 10^{-5} T - 7.455610^{-3} \tag{5}$$

$$\rho_g = (p + \rho_0) M_w / 8.314T \tag{6}$$

$$\sigma = 0.14783 \left(1 - \frac{T(p)}{T_c}\right)^{1.053} \tag{7}$$

Supplementary Section 2: System meshing

In the present system, the triangular mesh was used. As can be seen in the geometric shape of the system, the initial nucleus of the bubble was placed in the left corner, so to minimize the volume and calculations time, very fine grid was only used in this corner. A fine grid which

expanded by 2.5% (growth rate of 1.025) from the finer part of the computing domain was considered in the lower and upper right corner. The final specifications of the used mesh in this study, which was obtained after the mesh independence test, are presented in Supplementary Table 2.

Supplementary Table 2 Mapped mesh specifications

**Property**	**Type/Value**	**Property**	**Type/Value**
Types of Grids	Triangular	Grid Improvement Method	Regular
The Smallest Grid Size (Minimum Element Size)	0.000015	The Largest Grid Size (Maximum Element Size)	0.0002
Total Number of Elements	9623	Grid Bending Factor (Curvature Factor)	0.3
The Number of Degrees of Freedom	122134	Element Growth Rate	1.027

In this research work, the mesh independence test was investigated by evaluating characteristics such as the bubble departure diameter and the heat flux passing through the wall. The heat flux passing through the wall and the bubble departure diameter as a function of the number of elements in the grid are shown in Supplementary Fig. 2 (A) and (B) respectively. Based on the results, a mesh with 9623 elements was used as the optimal mesh in this study. In present work, an adaptive time step was used. Therefore, there is no need to check the independence of the time step, and the error rate is automatically checked at any time.

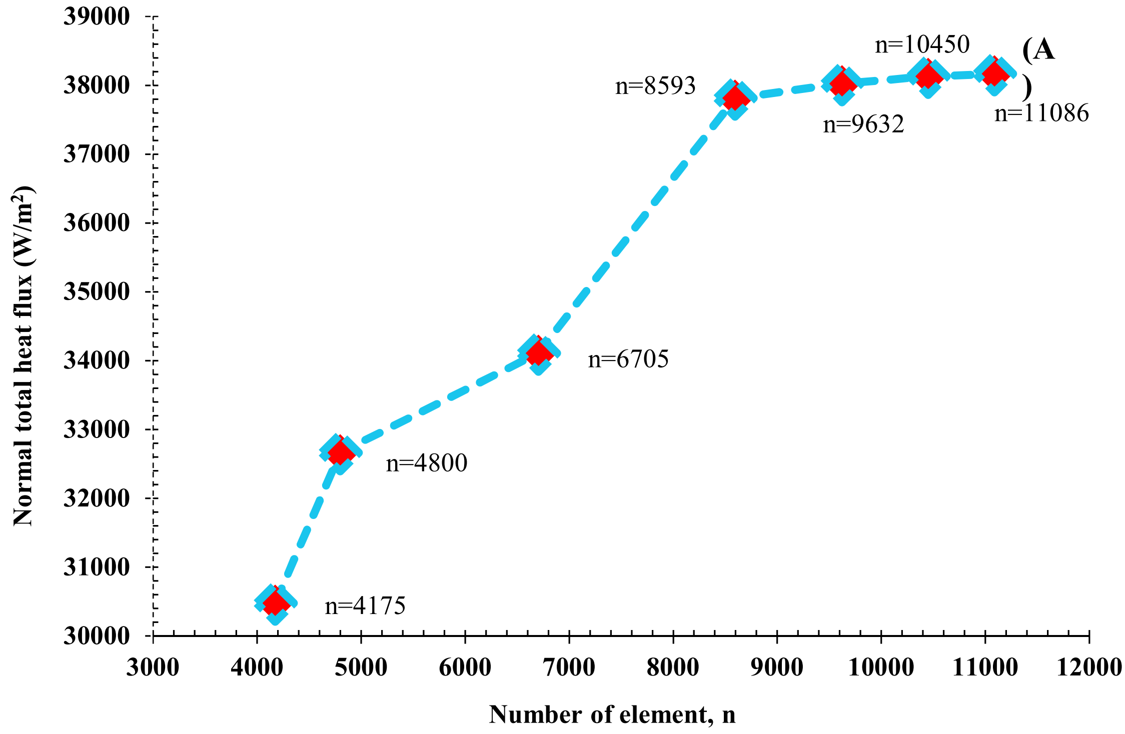

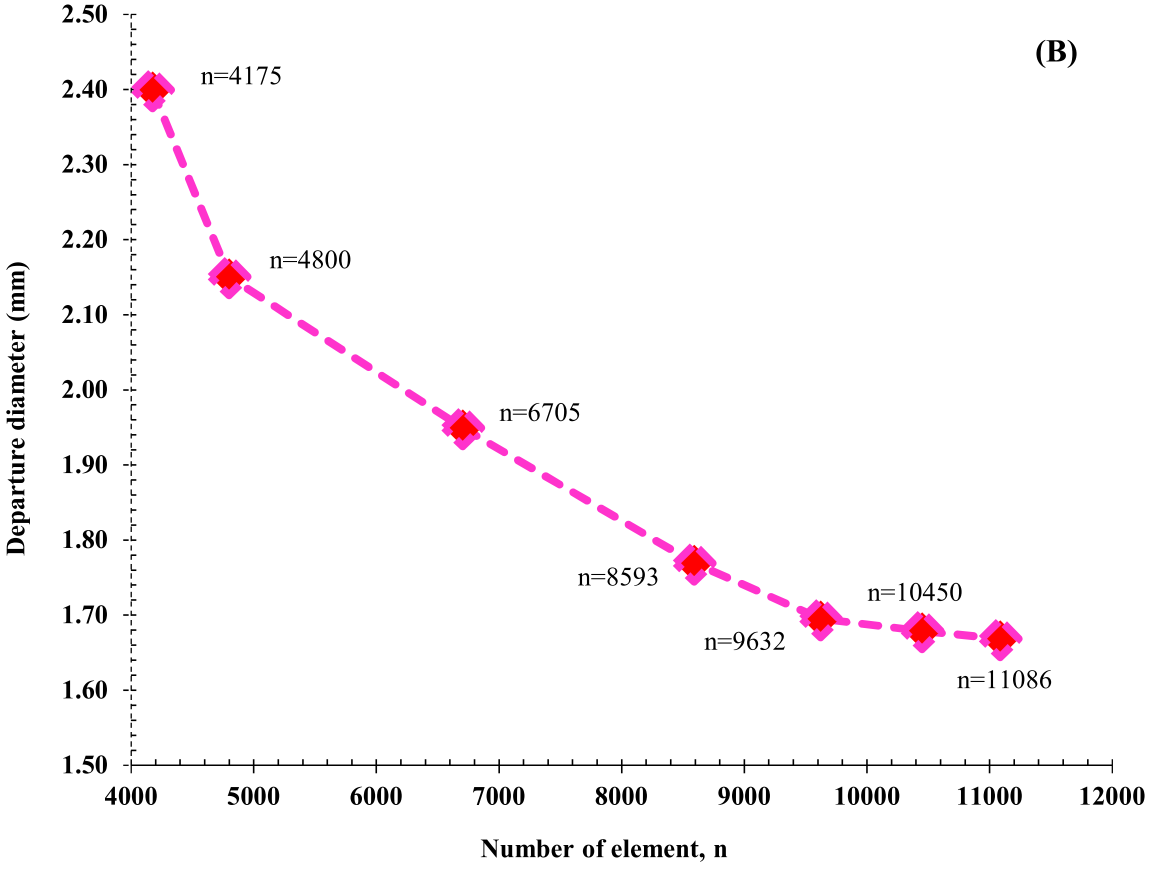

Supplementary Fig. 2 The mesh independence analysis (A) Heat flux changes versus the number of elements (B) Variations of the bubble departure diameter versus the number of elements.

Supplementary Section 3: The developed model initialization

Boiling is the formation of vapor bubbles on a hot solid surface. If the boiling is homogeneously performed on a perfectly smooth surface without any gaps and porosity, the solid surface will be completely covered with a layer of liquid. In this state, at a certain pressure, the surface temperature should reach the required amount for the bubble formation. A very high-temperature flux is required at the surface, which is determined by the amount of liquid heat transfer coefficient. But in reality, the surfaces are not completely smooth and have holes with different sizes depending on the material. The initial nucleus of steam is formed in these cavities. In fact, the tiny amount of trapped air in these cavities will be the initial cause of the vapor formation. The initial vapor nucleus is placed on the surface with an angle of 50° as shown Supplementary Fig. 3 (A). The quality of this vapor bubble is considered to be between 0.5 and 0.998 of the saturated vapor, after the initialization in the solution steps. Supplementary Fig. 3 (B) shows the volume fraction of the vapor on the cross-section of Supplementary Fig. 3 (A). The initialization is done only for the momentum and mass equations and does not include heat transfer equations. The time in the initialization is longer than the required time for the complete solution. The initial velocity is set to zero in both directions. Supplementary Fig. 3 (C) shows the relative permeability diagram in the cross section of Supplementary Fig. 3 (A).

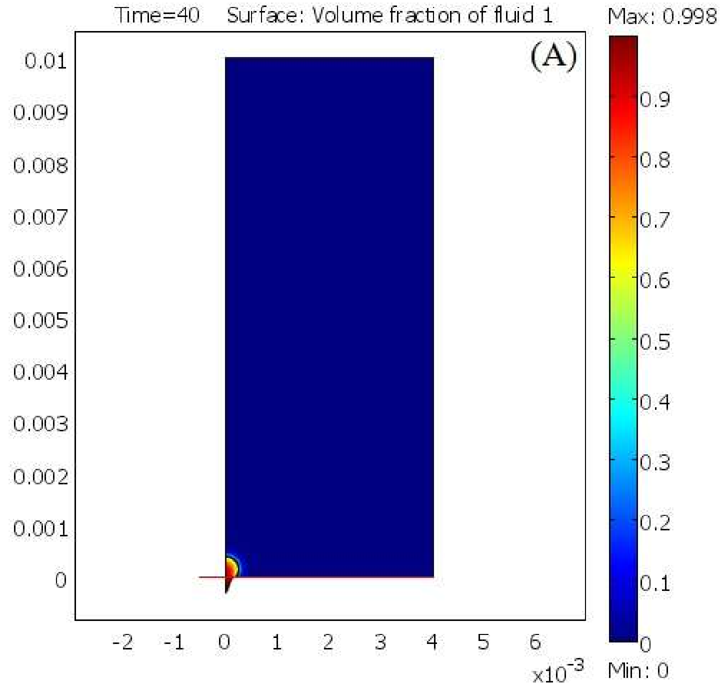

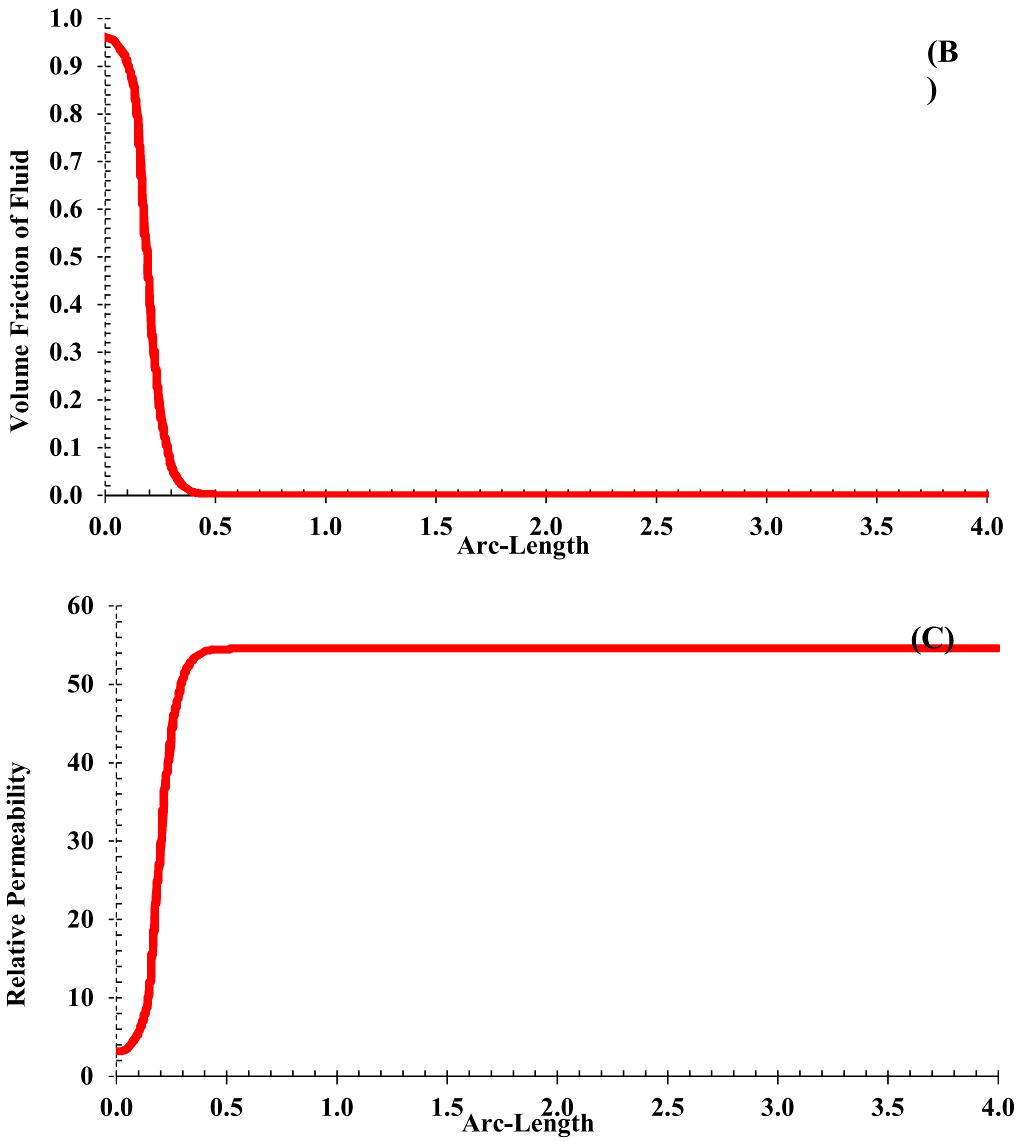

Supplementary Fig. 3 (A) Volume fraction of vapor after initialization. (B)The profile of the vapor volume fraction (computational domain cross section) (C) The profile of relative permeability (Computational domain cross-section)

Supplementary Section 4: Results and discussion (Additional explanation)

Effect of the temperature and velocity field on the bubble behavior

The velocity field for the two contact angles of 90° and 50° is presented in the step of pulling back of the bubble base and the bubble moving in the departure direction from the surface in Supplementary Fig. 4 (A-B). Moving the bubble base to the left can result in rotation of the fluid around the bubble in a clockwise and pulling the cooler saturated liquid on the hot wall. As can be seen in Supplementary Fig. 4 (A-B), this rotation is clearer for the larger contact angle and involves a larger volume of liquid. Therefore, by reducing surface wetting, the

bubble departure diameter becomes larger and a larger vortex is formed during the departure time around the vapor.

To compare the temperature field before the bubble departure, the dimensionless temperature isotherms (T^*) were given at intervals of 0.1 for two different contact angles of 50° and 90° in Supplementary Fig. 4 (C-D). Comparing the results of the contact angle of 90° with the contact angle of 50° indicates that the liquid rotation is more effective in turning away more volume of the superheated liquid from the wall. Also by the bubble departure from the surface, the rotation in a clockwise direction causes the saturated liquid vortex was dragged down to the end of the computational domain. This phenomenon leads to compression of the thermal boundary layer on the wall. Due to the presence of a stronger vortex at the larger contact angle, the bubble thermal boundary layer thickness is thinner. Thinner thermal boundary layer thickness increases the wall heat flux.

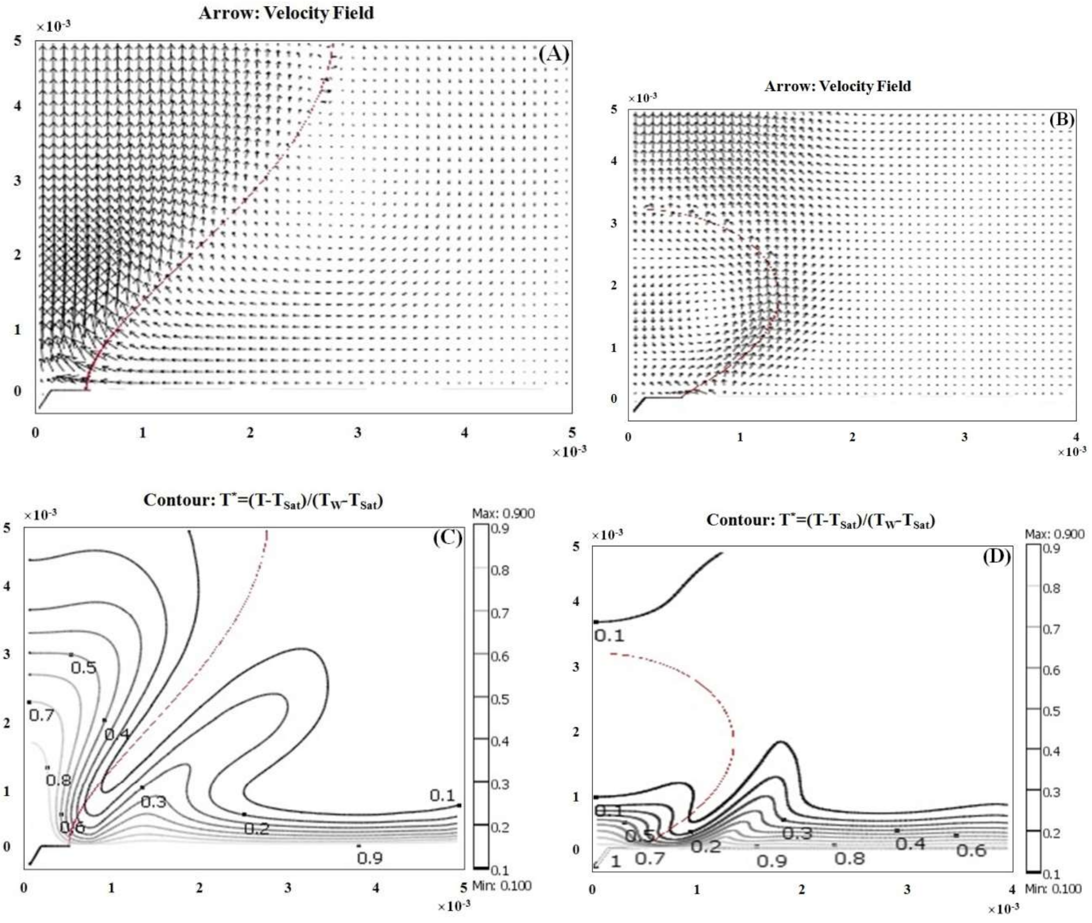

Supplementary Fig. 4 The velocity field near the bubble base in the departure stage (A) Contact angle of 90° (B) Contact angle of 50°. The temperature field near the bubble base in the departure stage (C) Contact angle of 90° (b) Contact angle of 50°

Heat (or enthalpy) of vaporization

In the relative boiling regime of isolated bubbles, nucleate boiling and natural convection both contribute to the heat transfer. By increasing the temperature above the saturation temperature, the fluid begins to change the phase, which is accompanied by the release of latent heat of evaporation. As can be seen in Supplementary Fig. 5 (A) and (B), the interface of the two-phases has a higher heat generation rate. The highest heat is at the junction of the bubble with the hot surface, which is the junction of the three phases. The highest temperature at the surface causes the phase change and higher heat generation rate. Supplementary Fig. 5 (C) shows the generation of heat at two different temperatures in the lines passing through the hot surface, 1 mm and 0.1 mm above the hot surface. The horizontal axis indicates the distance from the cylinder center with a radius of 4 mm. As can be seen, this heat has a value only in the narrow region where the phase change takes place. But, the generated heat in the vapor phase and liquid phase is zero in all three cases. In addition, the amount of the generated heat is maximum at higher temperatures for all cases.

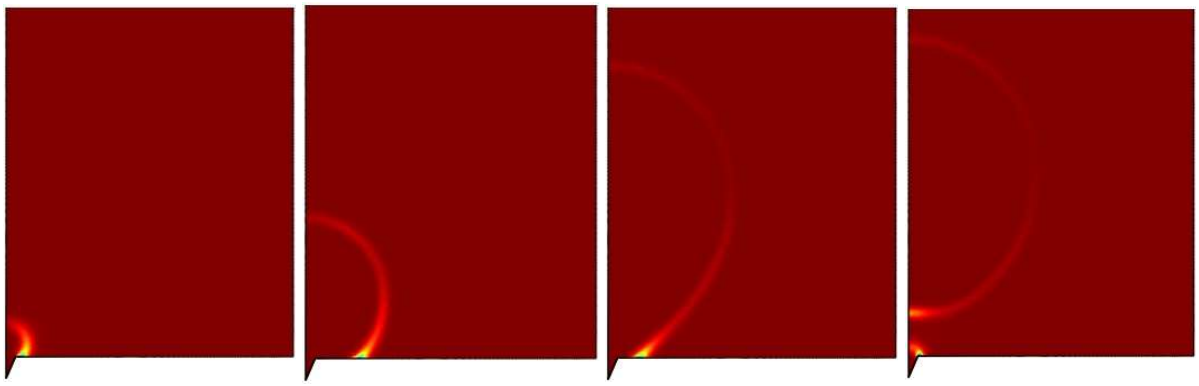

(A)

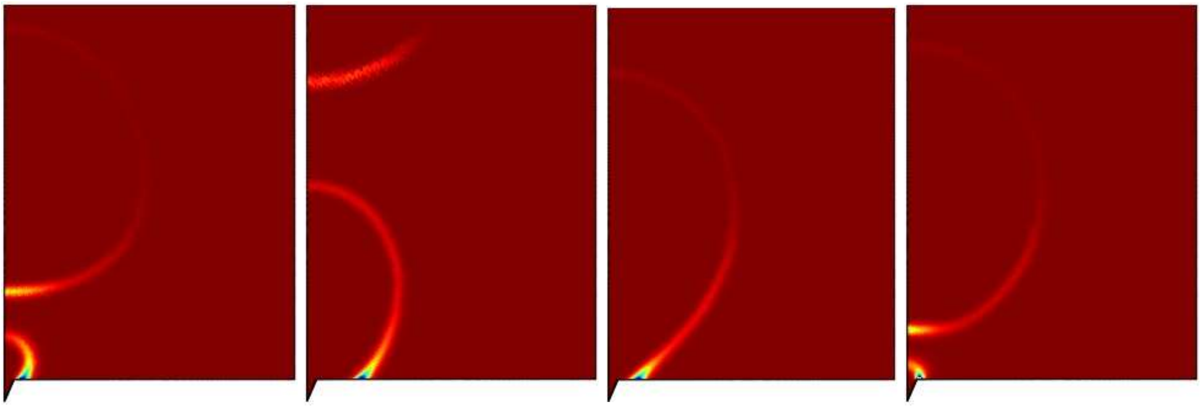

(B)

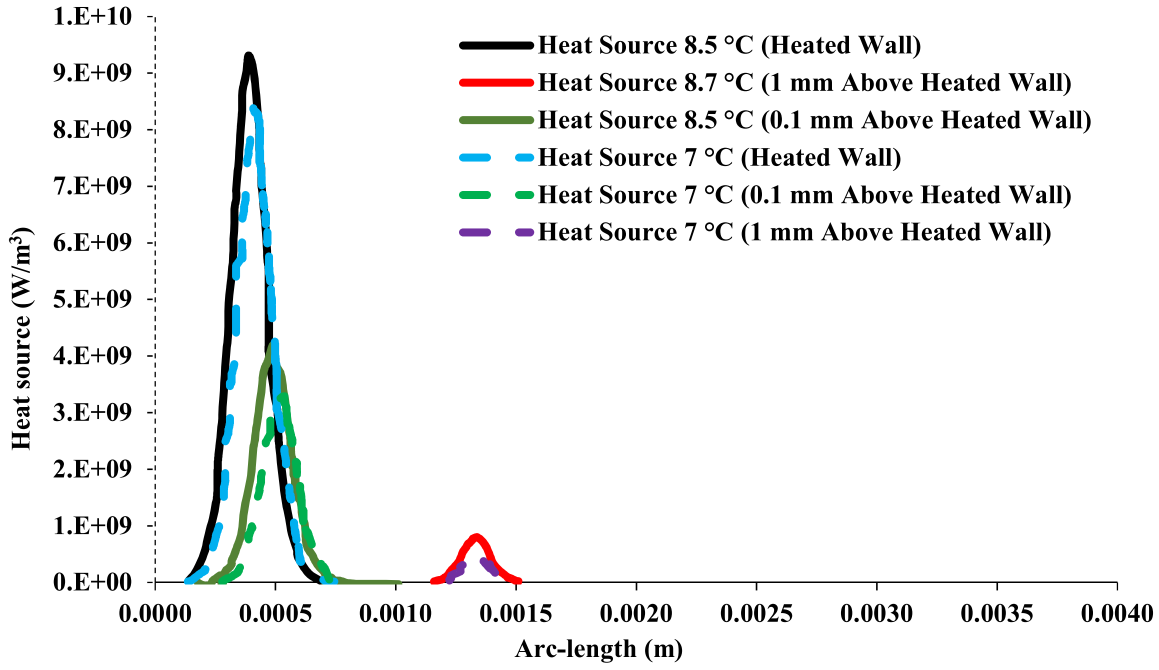

(C)

Supplementary Fig. 5. The Generated heat by evaporation (A) Temperature 7 K (B) Temperature 8.5 K (C) Profile of generated heat by evaporation in the lines passing through the hot surface, 1 mm and 0.1 mm above the hot surface for two temperatures

Interface length of consecutive bubbles

When the water starts to boiling, the surface area (length in two dimensions) of the interface increases and its value can be obtained by integration of δ on the computational domain based on Eq. 8:

$$A_s = \int_V \delta . dV \tag{8}$$

Since vapor is continuously released and departed from the surface, simultaneously, the generated vapor bulks are expanded and penetrated into the liquid free surface. It causes the fluctuation of surface area of the interface. Supplementary Fig. 6 (A) shows the interface length in the time interval of 1.2 s, where the 12 produced bubbles grow and depart from the surface. Also, the structure of the growing bubbles with a contact angle of 50° for the superheat with temperatures of 7 °C and 8.5 °C is given in Supplementary Fig. 6 (B) and (C), respectively. It should be noted that the first bubble has a longer growth time.

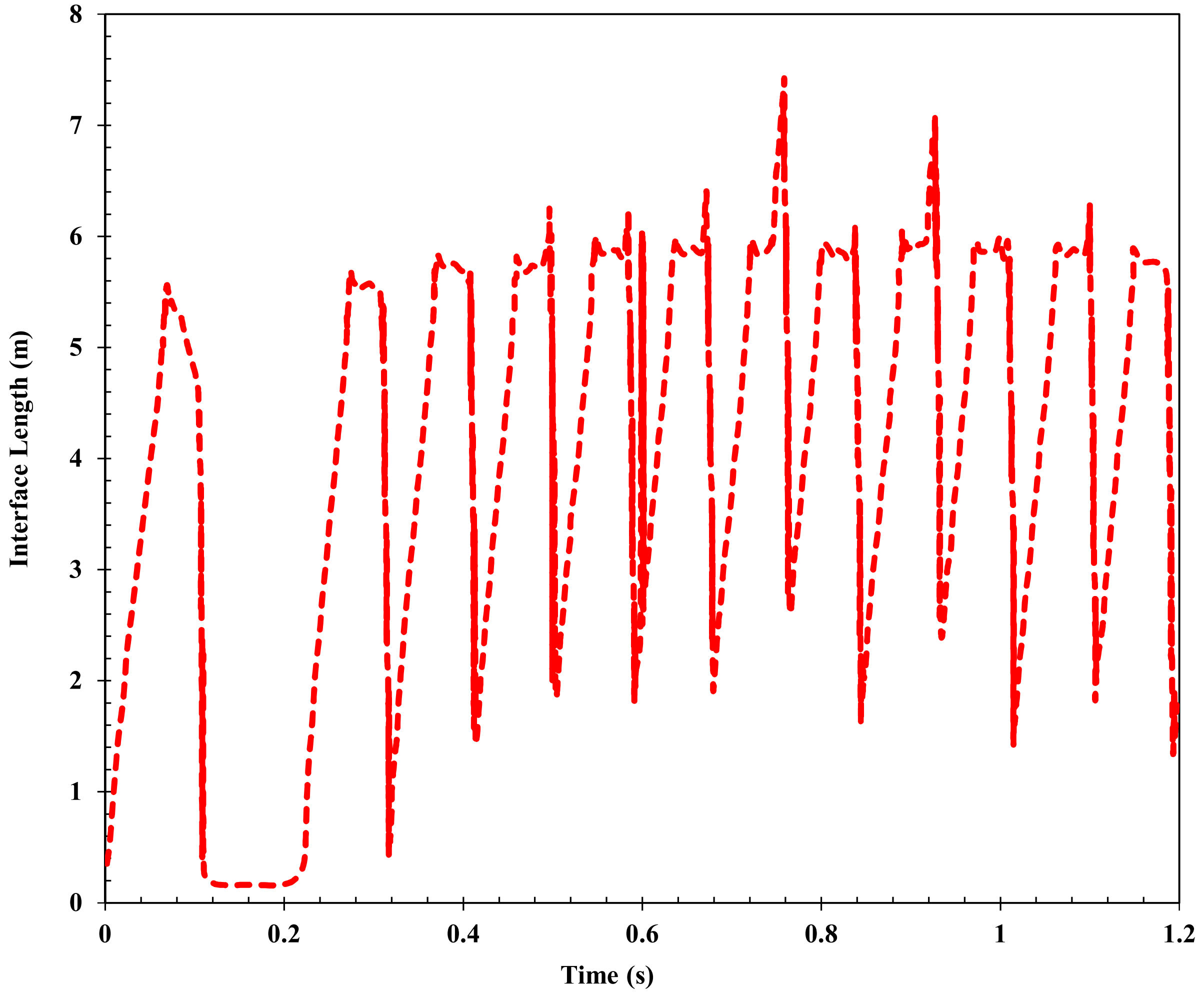

(A)

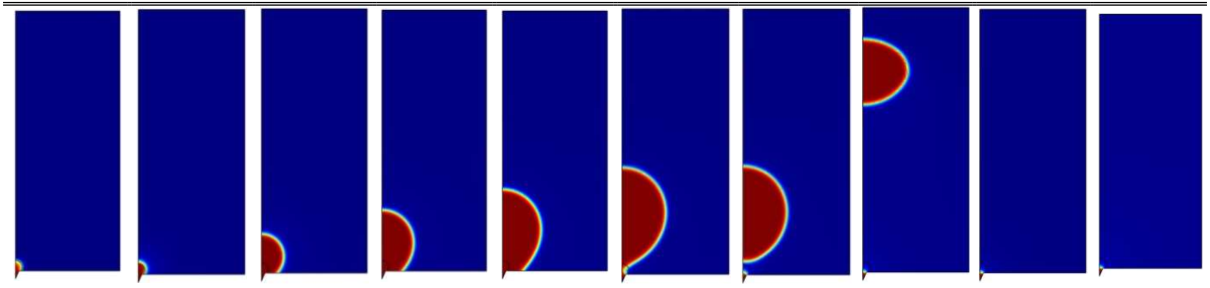

$t_0=0$	$t_0=0.46$	$t_0=1.46$	$t_0=2.46$	$t_0=3.46$	$t_0=4.4$	$t_0=4.46$	$t_0=6.46$	$t_0=8.46$	$t_0=10.46$

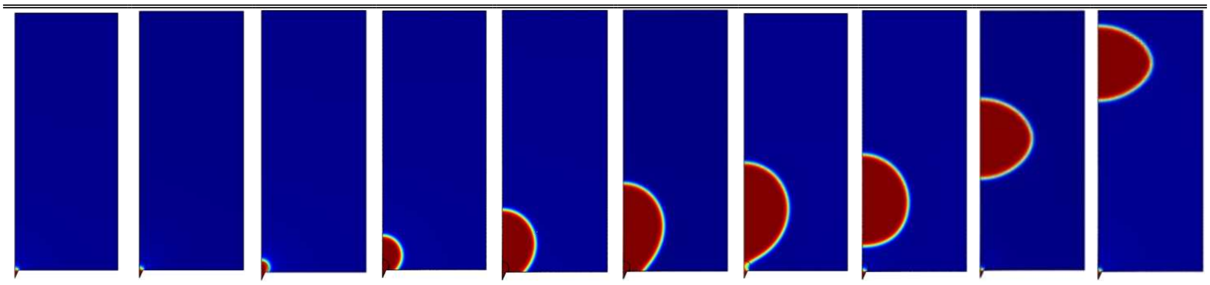

$t_0=12.46$	$t_0=13.46$	$t_0=13.9$	$t_0=14.46$	$t_0=15.46$	$t_0=16.46$	$t_0=17.25$	$t_0=17.46$	$t_0=18.46$	$t_0=19.46$

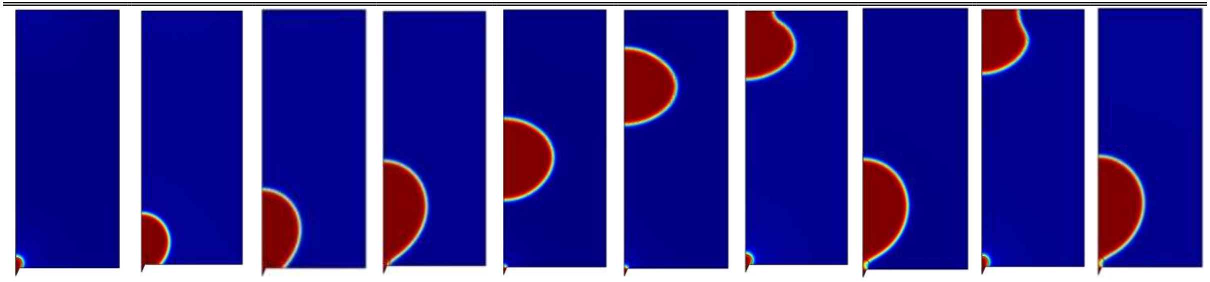

t_0=20.46	t_0=21.46	t_0=22.46	t_0=23.46	t_0=24.46	t_0=25.46	t_0=25.99	t_0=29.16	t_0=31.66	t_0=34.97

(B)

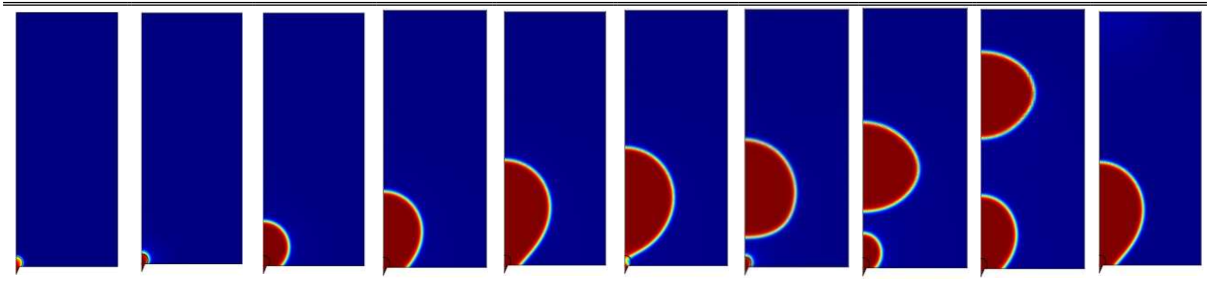

t_0=0	t_0=0.27	t_0=1.27	t_0=2.27	t_0=3.27	t_0=3.66	t_0=3.89	t_0=4.27	t_0=5.27	t_0=6.27

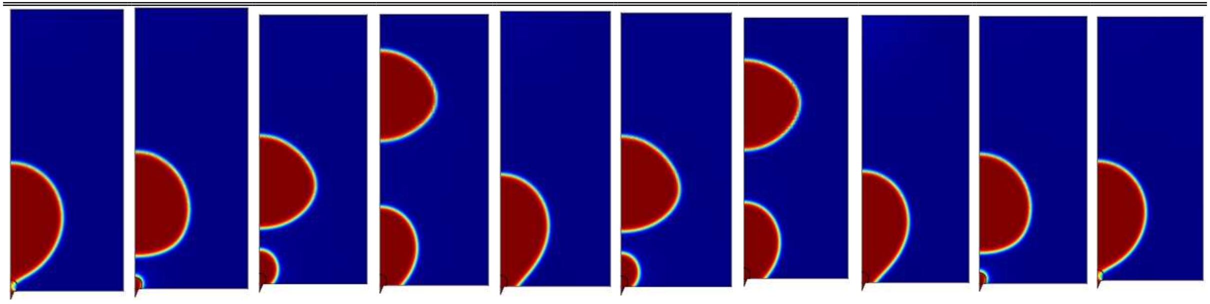

t_0=6.68	t_0=6.92	t_0=7.27	t_0=7.28	t_0=9.27	t_0=10.27	t_0=11.27	t_0=12.27	t_0=12.80	t_0=15.41

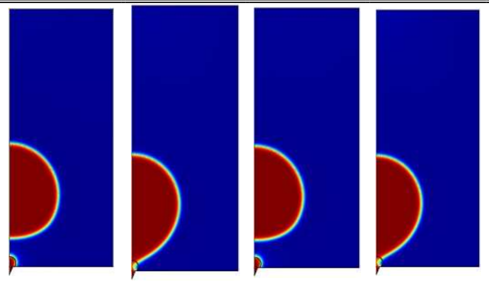

t_0=45.44	t_0=47.95	t_0=48.18	t_0=50.61

(C)

Supplementary Fig. 6 (A) Diagram of the length of the interface vapor-liquid and bubble growth in (B) Superheat 7°C and contact angle 50° (C) Superheat 8.5°C and contact Angle 50°

Total free energy of the system

In the boiling system, for the total free energy of the system, only the mixing energy part is considered and estimated by the Ginsburg-Landau function. Therefore, the following two definitions are used to obtain the free energy of the system:

$$F = A_s . \sigma \quad (15)$$

$$F = \int \left(\frac{1}{2} \lambda |\nabla \emptyset|^2 + \frac{\lambda}{4\varepsilon^2} (\phi^2 - 1)^2 \right) dV \quad (9)$$

The difference in the values of these two functions indicates that the phase-field model used in the present study is not suitable for estimating the system's free energy. Supplementary Fig. 7 (A) shows the total free energy of the system as a function of time for two different methods. In the beginning, two methods are well-matched, but as the growth and departure of the bubble periodically begin over time, a deviation occurs which can be ignored. Basically, when the bubbles diffuse within the liquid, "mass loss" occurs, which is due to the creation of an interface between two phases, and the mesh variability is not enough to accurately determine this effect. Supplementary Fig. 7 (B) shows the free energy for two methods when applying a voltage of 4,000 V. In this case, the obtained free energy using both equations have increased slightly, which can be attributed to the increase in the interface length and mixing energy due to the applying of an electric-field.

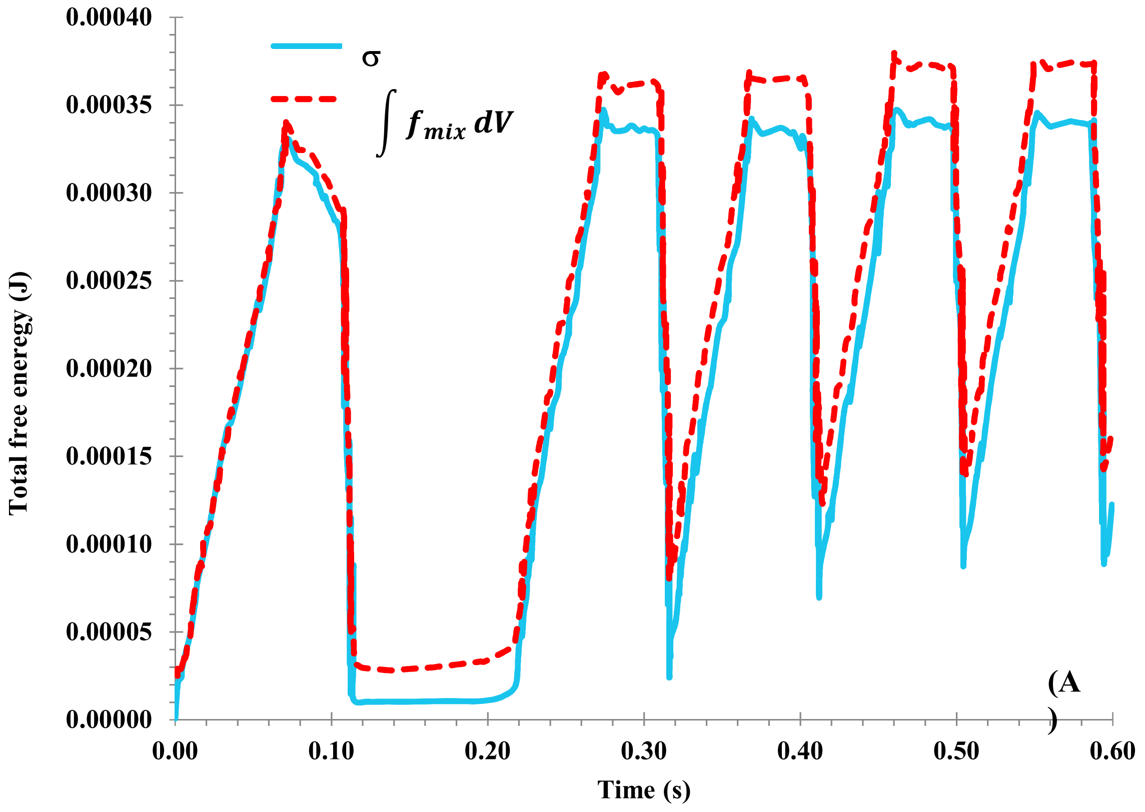

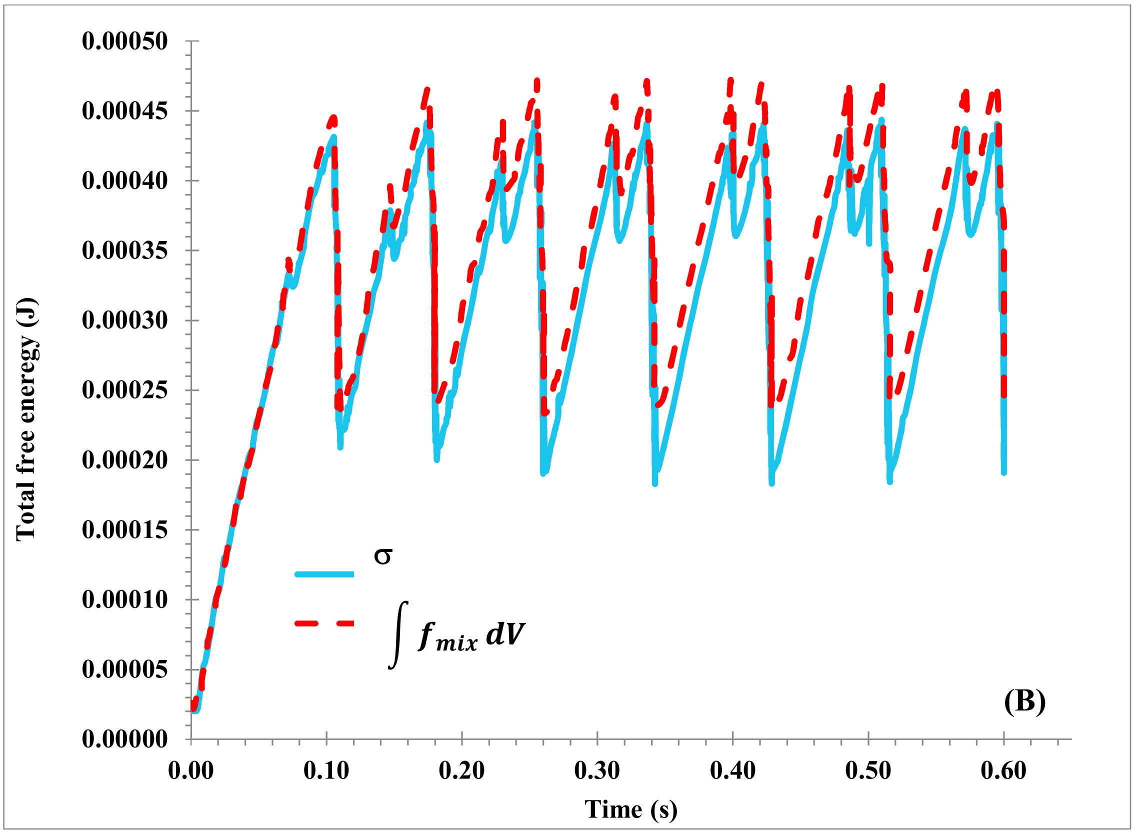

Supplementary Fig. 7 Diagram of the system free energy (A). Without applying electricalv (B). With applying electrical voltage [using Eq. (29) (solid Line) and Eq. (30) (dotted line)]

To determine the effect of the electric-field on the heat transfer rate, the heat flux diagram and the Nusselt dimensionless number are plotted for different voltages over a certain period of time in the main manuscript. In addition, the electro-hydrodynamic force changes the temperature field and the stream line near the hot wall and the side of the bubble. The electro-hydrodynamic force changes the temperature field near the hot wall by changing the velocity field, growth time, and bubble departure and size. Based on the presented results in Supplementary Fig. 8, by applying an electric field and increasing the applied voltage, the thickness of the thermal boundary layer is reduced and the isothermal lines near the wall and towards the end of the computing domain are compressed. Reducing thickness of the thermal boundary layer causes increasing the heat flux passing through the hot surface.

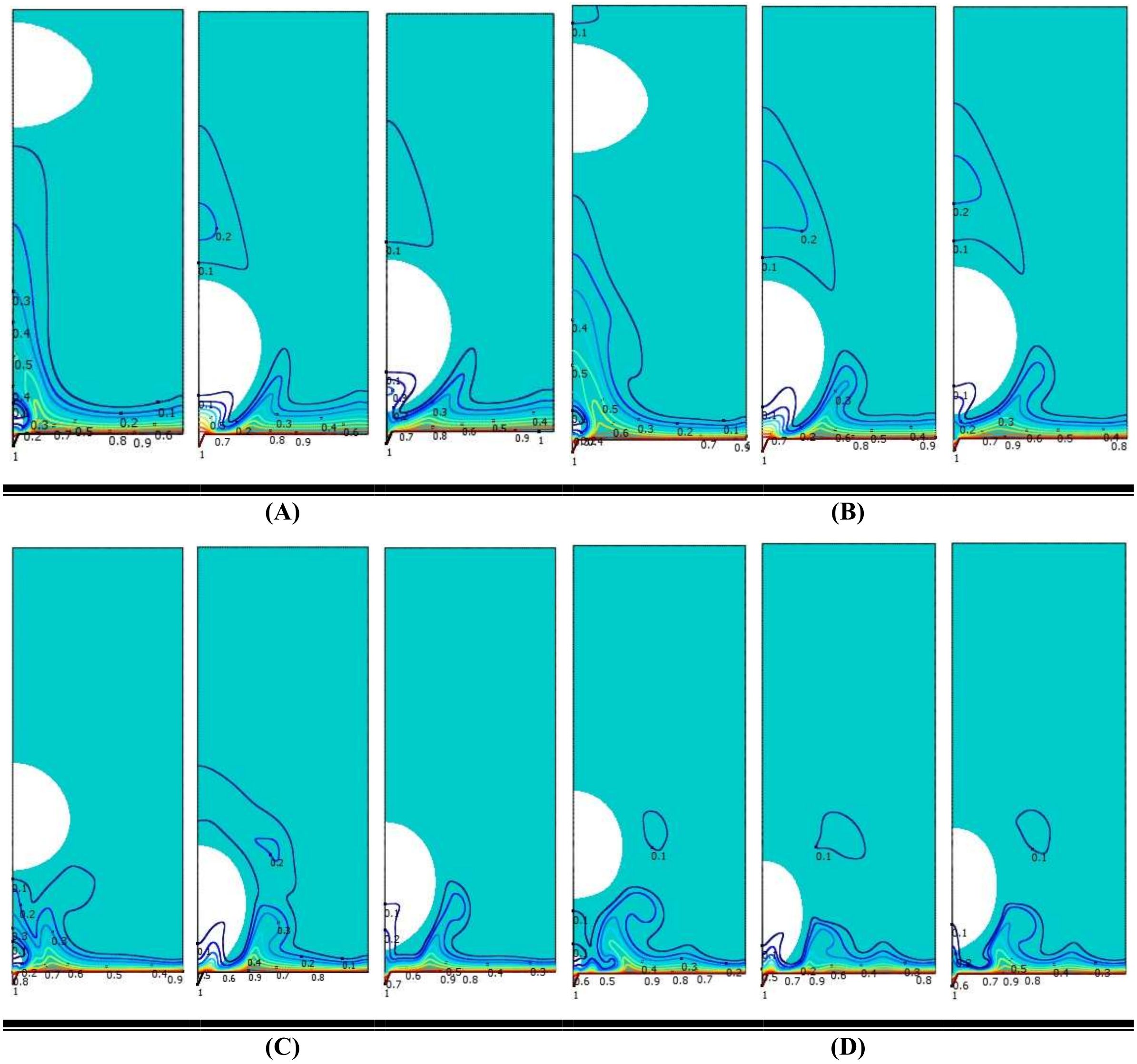

Supplementary Fig. 8 Displays changes of the temperature field in the vicinity of a hot wall for different voltages for superheat 7°C and contact angle 50° (a) Zero voltage (b) V = 1000v (c) V = 2000v (d) V = 3000v

- **Nomenclature**

PFM: Phase-Field Method
LBM: Lattice Boltzmann Method
VOF: Volume of Fluid Method
T^*: Dimensionless Temperature
ΔT_{sup}: Superheated Rate of the Wall, [°C]
f_{tot}: Total Free Energy Density, [J/m^3]
f_{ext}: Determined Free Energy, [J/m^3]
δ: Thermal Boundary Layer Thickness, [μm]
ΔV: Electrical Potential Difference
V_f: Volume Fraction for Vapor or Liquid
δ: Length of the Interface Between Two Phases
u_{int}: Velocity in two-Phase Interface, [m/s]
k: Thermal Conductivity Coefficient, [W/(m.K)]
C_p: Heat Capacity of the Vapor Phase, [kJ/(kg.K)]
k_v: Heat Transfer Coefficient of the Vapor Phase, [W/(m^2.K)]
$\Delta H_{l.v}$: Vapor Enthalpy, [KJ/mol]
M_w: Molecular Mass of Vapor, [Da]
F: Electrostatic Force (Divergence of Maxwell Stress Tensor)

l_0: Characteristic Length
t_0: Characteristic Time
$\dot{m}$: Rate of Vapor Production
G: Chemical Potential, [Pa]
u_v: $Vapor\ Velocity$, [m/s]
u_0: Fluid Velocity, [m/s]
f: Departure Frequency, [1/s]
E: Electric Field
u_l: $Liquid\ Velocity$, [m/s]
t_g: Growth Time, [ms or s]
B: Magnetic Field
S: Closed Surface
n: Unit Normal Vector
LSM: Level Set Method
t_w: Residence Time, [s]
q: Heat Flux, [W/m^2]

- **Greek symbols**

ε: Absolute Permittivity of Space Between Charges
ε_r: Relative Permittivity or Dielectric Constant of Medium
ε: Capillary Width(Thickness of the Interface)
χ: Mobility Tuning Parameter , [m.s/kg]
σ: Surface Tension Coefficient, [N/m]
γ: Mobility Parameter , [m^3.s/kg]

λ_ε = Permittivity Ratio
λ: Mixing Energy Density
ε_0: Vacuum Permittivity, [F/m]
ϕ: Phase − Field Variable
α: Fluid Diffusion Coefficient
ρ: Density of the Fluid,

[kg/m^3]

F_σ = Surface Tension, [N/m^3]

F_e = Electric Field Force, [N/m^3]

g = Gravitational Acceleration, [m/s^2]

λ_ρ = Density Ratio

υ : Kinematic Viscosity of the Fluid, [Cm^2/s]

λ_μ = Viscosity Ratio

β: Volume Thermal Expansion Coefficient of the Fluid(at Constant Pressure)

- **Dimensionless Number**

Grashof Number: $Gr = \frac{g\beta\Delta T_{sup} l_0^3}{\upsilon^2}$

Rayleigh number: $Ra = Gr.Pr = \frac{g\beta\Delta T_{sup} l_0^3}{\vartheta\alpha}$

Prandtl number: $Pr = \frac{\vartheta}{\alpha}$

Fluid Diffusion Coefficient: $\alpha = \frac{k}{\rho C_p}$

Volume Thermal Expansion Coefficient of the Fluid: $\beta = -\frac{1}{\rho}\left(\frac{\partial\rho}{\partial T}\right)_p \rightarrow$

(for water is 0.000749)

Bo_e = Electric Bond Number

Eo = Eotvos Number

M = Morton Number

Nusselt number: $Nu = l_0 q/k\Delta T_{sup}$

- **Subscripts and superscripts**

v: Vapor

l: Liquid

Reference

1. Ghiaasiaan, S.M., *Two-phase flow, boiling, and condensation: in conventional and miniature systems*. 2007: Cambridge University Press.
2. Incropera, F.P., et al., *One-dimensional, steady-state conduction.* Fundamentals of Heat and Mass Transfer, Third Edition", Incropera, FP and DeWitt, DP, John Wiley & Sons, New York (1990a), 2007: p. 96-99.
3. Bird, R., W. Stewart, and E. Lightfoot, *Transport Phenomena, John Wiley & Sons, New York, NY, USA.* 2002.
4. Bergman, T.L., et al., *Introduction to heat transfer*. 2011: John Wiley & Sons.
5. Takada, N., M. Misawa, and A. Tomiyama. *A phase-field method for interface-tracking simulation of two-phase flows*. in *Fluids Engineering Division Summer Meeting*. 2005.

6. Yue, P., et al., *Phase-field simulations of interfacial dynamics in viscoelastic fluids using finite elements with adaptive meshing.* Journal of Computational Physics, 2006. **219**(1): p. 47-67.
